# Insights Into the Impact of Small RNA SprC on the Metabolism and Virulence of *Staphylococcus aureus*

**DOI:** 10.3389/fcimb.2022.746746

**Published:** 2022-02-23

**Authors:** Jingwen Zhou, Huanqiang Zhao, Han Yang, Chunyan He, Wen Shu, Zelin Cui, Qingzhong Liu

**Affiliations:** ^1^Department of Clinical Laboratory, Shanghai General Hospital, Shanghai Jiaotong University School of Medicine, Shanghai, China; ^2^Obstetrics and Gynaecology Hospital, Fudan University, Shanghai, China; ^3^The Shanghai Key Laboratory of Female Reproductive Endocrine-Related Diseases, Shanghai, China; ^4^Department of Clinical Laboratory, Shanghai Municipal Hospital of Traditional Chinese Medicine, Shanghai University of Traditional Chinese Medicine, Shanghai, China

**Keywords:** *Staphylococcus aureus*, SprC, small RNA, transcriptome, regulation role, metabolomics

## Abstract

**Aim:**

Our previous proteomic analysis showed that small RNA SprC (one of the small pathogenicity island RNAs) of *Staphylococcus aureus* possesses the ability to regulate the expression of multiple bacterial proteins. In this study, our objective was to further provide insights into the regulatory role of SprC in gene transcription and metabolism of *S. aureus*.

**Methods:**

Gene expression profiles were obtained from *S. aureus* N315 wild-type and its *sprC* deletion mutant strains by RNA-sequencing (RNA-seq), and differentially expressed genes (DEGs) were screened by R language with a |log2(fold change)| ≥1 and a false discovery rate (FDR) ≤ 0.05. Gene Ontology (GO) and Kyoto Encyclopedia of Genes and Genomes (KEGG) pathway analysis were carried out to understand the significance of the DEGs. The quality of RNA-seq was further verified by quantitative real-time PCR (qRT-PCR), mRNA target prediction, metabolomics analysis and transcript-level expression analysis of genes of *sprC* complementation strain.

**Results:**

A total of 2497 transcripts were identified, of which 60 transcripts expressions in *sprC* knockout strain were significantly different (37 up-regulated and 23 down-regulated DEGs). GO analysis showed that the functions of these DEGs were mainly concentrated in the biological process and molecular function related to metabolism and pathogenesis, and a higher number of genes were involved in the oxidation-reduction process, catalytic activity and binding. KEGG pathways enrichment analysis demonstrated that metabolism and pathogenesis were the most affected pathways, such as metabolic pathways, biosynthesis of secondary metabolites, purine metabolism, fructose and mannose metabolism and *S*. *aureus* infection. The qRT-PCR results of the DEGs with defined functions in the *sprC* deletion and complementation strains were in general agreement with those obtained by RNA-seq. Metabolomics analysis revealed 77 specific pathways involving metabolic pathways. Among them, many, such as metabolic pathways, biosynthesis of secondary metabolites and purine metabolism, were consistent with those enriched in the RNA-seq analysis.

**Conclusion:**

This study offered valuable and reliable information about the regulatory roles of SprC in *S*. *aureus* biology through transcriptomics and metabolomics analysis. These results may provide clues for new potential targets for anti-virulence adjuvant therapy on *S. aureus* infection.

## Introduction

*Staphylococcus aureus* (*S. aureus*) is a common pathogenic Gram-positive bacterium that produces a plethora of virulence factors (Tong et al., 2015; Lee et al., 2018). *S. aureus* can get access to the tissues or the bloodstream under the skin and cause severe infections through wounds on the skin or mucosa (Lee et al., 2018). Besides, its pathogenicity is closely related to the adaptability of this pathogen in the host, which results from changes in bacterial metabolism and virulence (Balasubramanian et al., 2017; Richardson, 2019). Therefore, studying the regulatory mechanism underlying the virulence and metabolism of *S. aureus* is of infinite significance.

Previous studies showed that small regulatory RNAs (sRNAs) help *S. aureus* adapt to the environment quickly, which has a strong relationship with bacterial pathogenicity (Caldelari et al., 2013; Desgranges et al., 2019; Georg et al., 2020). Hundreds of sRNAs have been identified, of which 8 small pathogenicity island (SaPIn) RNAs (SprA-G and X) were verified and several were studied in detail (Pichon and Felden, 2005; Eyraud et al., 2014). For example, SprA1AS can intercept the translation initiation signal (Sayed et al., 2011), SprD suppresses the expression of Sbi protein and promotes the virulence of *S. aureus* (Chabelskaya et al., 2010), and SprX is involved in glycopeptides resistance and the regulation of virulence factors (Eyraud et al., 2014; Kathirvel et al., 2016). Gene *sprC* is located next to gene *lukD* and *lukE* on SaPIn3 (Pichon and Felden, 2005), and is named srn_3610 in the staphylococcal regulatory RNA database established by Sassi et al. (2015). Le Pabic et al. (2015) reported that SprC weakened the virulence and leukocytic phagocytosis of *S. aureus*, which in turn attenuated the pathogenicity of the bacteria. In an early study, we reported the significantly influence of SprC on the expressions of proteins involving in metabolic process, cellular process, biological modulation and catalytic activity in *S. aureus* by proteomics analysis (Zhao et al., 2017). In addition, it was reported that sRNAs modulate various targets, including RNA, protein and DNA, thereby controlling gene transcription and translation, mRNA stability, and DNA maintenance or silencing (Diallo and Provost, 2020). To further understand the influence of SprC on bacterial gene expression and metabolism, in this study, we analyzed the differences in the transcriptional and metabolic profiles in the *sprC* deletion mutant of *S. aureus* N315 by using RNA sequencing (RNA-seq) and liquid chromatography tandem-mass spectrometry (LC-MS/MS), respectively. The results will enrich the knowledge about the physiological regulation networks of *S. aureus*.

## Materials and Methods

### Strains, Plasmids and Culture Conditions

Bacterial strains used in this study were *S. aureus* N315, N315Δ*sprC* (*sprC* deletion mutant) (Zhao et al., 2017), and *S. aureus* R4220. The plasmid pOS1 was a gift from Associate Professor Qian Liu. Bacteria were cultured at 37°C in tryptic soy broth medium (TSB; Oxoid), rotating at 200 revolutions per minute under aerobic conditions, with a volume to tube ratio of 1/3 according to our early study (Zhao et al., 2017).

### Total RNA Extraction and Qualification

Bacterial cultures that grew for 4 h to early logarithmic growth phase (OD_600_ = 0.5) were centrifuged at 13,000×g for 10 min at 4°C. The cell pellets were resuspended in 1 ml of TE buffer (10 mM Tris HCl, 1 mM EDTA, pH 8.0) supplemented with lysostaphin (1 mg/ml, Sangon Biotech) and proteinase K (20 mg/ml, TaKaRa), and incubated for 1 h at 56°C. Total RNA extraction was performed using a MiniBEST Universal RNA Extraction Kit (TaKaRa) according to the manufacturer’s protocol, including an optional on-column DNase treatment procedure (TianGen). RNA degradation and contamination were detected using 1% agarose gel electrophoresis. The purity and concentration of RNA were analyzed by a Nanodrop spectrometer (Thermo Fisher Scientific). The integrity of RNA was precisely measured using the RNA Nano 6000 Assay Kit, and quantified by the Agilent 2100 bioanalyzer (Agilent Technologies Inc.).

### RNA Sequencing (RNA-Seq) Library Construction

Bacterial mRNA was isolated with the MICROB*Express*™ kit (Invitrogen) by removing ribosomal RNA (rRNA) from total RNA according to the manufacturer’s instructions. The mRNA was fragmented and reverse transcribed with random hexamer primers (5’-d(NNNNNN)-3’ (N=G, A, T or C)) (Thermo Fisher Scientific) by using the Superscript™ Double-Stranded cDNA synthesis kit (Invitrogen). After repairing the cDNA ends using NEBNext End repair/dA-tailing module (New England Biolabs), sequencing adapter ligation was performed. Then, polymerase chain reaction (PCR) was carried out to generate the cDNA library. AMPure XP beads (Beckman Coulter Inc.) were utilized for purification after each enzymatic reaction. Following the construction of the cDNA library, preliminary quantification and the detection of the insert size were conducted by qubit 2.0 (Life Tech Invitrogen) and Agilent 2100, and the effective concentration of the library (> 2 nM) was accurately quantified by quantitative real-time PCR (qRT-PCR) to ensure the quality of the library. The cDNA library was established using NEBNext^®^ UltraTM II RNA Library Prep Kit (New England Biolabs).

### RNA Sequencing and Bioinformatics Analysis

The prepared cDNA Library was sequenced on Illumina HiSeq 4000 sequencing platform (Illumina) to generate 2 × 150 bp paired-end reads. Quality of RNA-seq data was comprehensively evaluated using RseQC package (version 2.6.3) (Wang et al., 2012). Raw data were aligned to the *S. aureus* NCTC 8325 genome sequence (GenBank accession number NC_007795). The quantitative expression level of each gene was represented by expected number of fragments per kilobase transcript sequence per million base pairs sequenced (FPKM), which was calculated using Cufflinks software (version 2.2.1). DESeq2 v 1.10.1 package was used to identify differentially expressed genes (DEGs) (with a |log_2_(fold change)| ≥1 and a false discovery rate (FDR) ≤ 0.05). Kyoto Encyclopedia of Genes and Genomes (KEGG) pathway enrichment analysis and Gene Ontology (GO) functional annotation on the DEGs were conducted based on the KEGG and GO databases (R package GOseq v 1.18) (Ashburner et al., 2000; Kanehisa and Goto, 2000).

### Quantitative Real-Time Polymerase Chain Reaction for DEGs Verification

In order to appraise the RNA-seq data, quantitative real-time polymerase chain reaction (qRT-PCR) was performed to quantify the level of the DEGs. Total RNA was extracted and reverse transcribed into cDNA using the Takara RNA PCR kit (AMV) ver.3.0 kit (TaKaRa), according to the manufacturer’s protocol. Then qRT-PCR was carried out in a 20 μL volume using SYBR Premix Ex Taq™ (Tli RNaseH Plus) kit (TaKaRa) as recommended by the manufacturer. Primers used are listed in **Supplementary Table 1**. Thermal cycling profile was as follows: 95°C for 30 sec, followed by 40 cycles of 95°C for 5 sec, 60°C for 34 sec; and 95°C for 15 sec, 60°C for 1 min, and 95°C for 15 sec. The *16S rRNA* was used as the reference gene for normalization. Three independent experiments were run in triplicate. Relative gene expression was assessed by the 2^-ΔΔCT^ method (Yang et al., 2020). Melting curve analysis was performed for gene amplification to analyze the primer efficiency.

In order to further confirm the influence of SprC on the expressions of DEGs, a complementation strain of *sprC* was constructed. Briefly, using DNA from strain N315 as a template, *sprC* gene was amplified by PCR with primer *sprC*-up-Nhel (CCGGCTAGCAAGTATTGAAAAATAAAATATTT) and *sprC*-down-BamHI (CGCGGATCCAACATATATATATTTACTATGAAC). SprC complementation plasmid was generated by cloning the *sprC* gene into the vector pOS1. The recombinant plasmid pOS1-*sprC* was first transferred into *S. aureus* RN4220 and then transformed into strain N315Δ*sprC via* electroporation (electrotransformation conditions: 1.8 kv, 2.5 ms). According to the methods described above, qRT-PCR was performed to check whether the expression levels of the DEGs (10 randomly selected genes) in the complementation strain (named N315Δ*sprC*-C) were backfilled to those in the wild-type N315 strain.

### Prediction of Binding Between DEGs-Transcribed mRNA and SprC

To validate that the mRNAs transcribed by DEGs could act as the targets of SprC, we calculated the thermodynamic stability of SprC:mRNA duplex using a bioinformatics tool “standalone algorithm RNAhybrid” on Bielefeld Bioinformatics Service website (https://bibiserv.cebitec.uni-bielefeld.de/). On this website, the molecular free energy (Mfe, kcal/mol) between the two molecules can be obtained. According to the description of Akhtar (Akhtar et al., 2019), a value of Mfe less than 0 indicated that SprC and mRNA could bind spontaneously with a good affinity.

### Metabolomic Analysis

Strain N315 and N315Δ*sprC* were collected at logarithmic growth phase and centrifuged at 14,000×g for 10 minutes at 4°C. The cell pellets were washed with phosphate-buffered saline (PBS) twice, then flash-frozen in liquid nitrogen, and stored at -80°C. The collected samples were thawed on ice, and metabolites were extracted by vortexing with 50% methanol buffer for 1 min, and incubating at room temperature for 10 min. The extraction mixtures were stored overnight at -20°C, then centrifugated for 20 min at 4,000×g. The supernatants were transferred into new 96-well plates and stored at -80°C prior to metabolomic analysis. The data of non-targeted metabolomic profiling were assembled in both positive ion (pos) and negative ion (neg) modes using LC-MS/MS technique (high-resolution mass spectrometer (Q Exactive), Thermo Fisher Scientific) to probe into the metabolomic compositions and their biofunctions (data from both groups, 6 biological repeats for each). Based on partial least squares method-discriminant analysis (PLS-DA) and variable importance in projection (VIP) value, the fold changes were used to identify differential metabolites. The differential ion defined needed to meet 3 conditions: (1) ratio ≥ 2 or ≤ 1/2; (2) *P*-value ≤ 0.05; (3) VIP ≥ 1. Compound Discoverer 3.1.0 software (Thermo Fisher Scientific) was used to identify statistically different metabolites. KEGG pathway database was utilized to annotate the different metabolites and exhibit the pathways with differential metabolite enrichment.

### Statistical Analysis

All statistical analyses were performed with SAS 9.3 for Windows software (SAS Institute Inc.) and R language. Data comparisons were performed using Student’s *t*-test. A *P*-value < 0.05 was considered a statistically significant difference.

## Results

### General Features of the Transcriptome Profile

Six cDNA libraries constructed based on samples of *S. aureus* N315 and N315Δ*SprC* (3 for each) were sequenced. The gene expression results of the three biological replicates obtained from the RNA-seq were highly consistent, which was more conducive to the subsequent transcriptome analysis. A total of 50,637,714 raw reads were generated. After quality control, 47,575,359 reads (24,279,637 and 23,295,722 for wild-type and knockout groups, respectively) were produced. The quality indicators of Q20 and Q30 were > 98% and > 95%, respectively, suggesting successful sequencing of the *S*. *aureus* transcriptome. Then the qualified reads were utilized for mapping to the reference genome of *S. aureus* NCTC 8325 for subsequent analysis. A detailed analysis of the RNA-seq on the six samples is represented in **Table 1**.

**Table 1:** The RNA-seq data after quality checking for 6 samples of *S. aureus* N315 and N315Δ*sprC*.

Sample	R1_reads	R1_bases	R2_reads	R2_bases	Q20 (%)	Q30 (%)
N315 T1	6,457,888	957,797,722	6,457,888	922,459,338	98.56	95.49
N315 T2	10,288,915	1,526,436,465	10,288,915	1,472,299,024	98.60	95.59
N315 T3	7,532,834	1,117,705,847	7,532,834	1,077,549,218	98.60	95.62
N315Δ*sprC* T4	9,952,252	1,476,521,194	9,952,252	1,433,511,080	98.75	95.99
N315Δ*sprC* T5	6,771,510	1,004,794,812	6,771,510	974,348,409	98.70	95.87
N315Δ*sprC* T6	6,571,960	974,229,014	6,571,960	943,416,970	98.64	95.69

The RNA-Seq data for 6 samples of S. aureus N315 and N315ΔsprC. Each sample yielded two sequences forward and reverse end sequences, described as R1 and R2 ends respectively. This table presents the data after quality control of the bases using Trimmomatic. Q20 (%) means the sequencing error rate of the base was less than 1%; Q30 (%) means the sequencing error rate of the base was less than 0.1%. Tn, sample number.

### Analysis of DEGs

**Figure 1:**
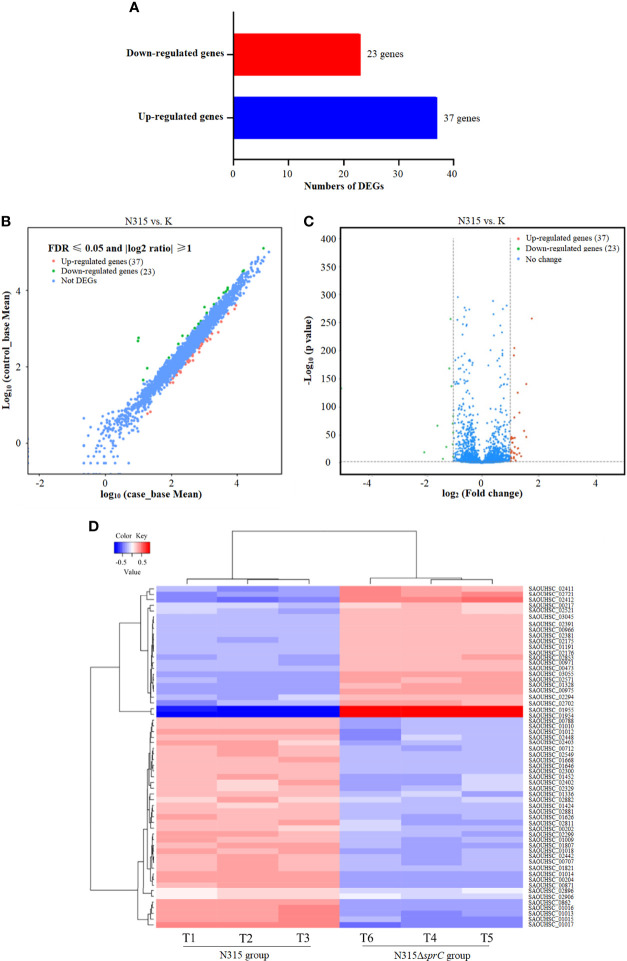
RNA-seq analysis of DEGs between wild-type *S. aureus* N315 and its *sprC* knockout mutant (N315Δ*SprC*). DESeq2 v 1.10.1 package was used to identify the DEGs (with a |log_2_(fold change)| ≥1 and a false discovery rate (FDR) ≤ 0.05). **(A)** A bar graph visualizing the number of up-regulated (blue color histogram) and down-regulated (red color histogram) genes. The x-axis indicates gene number. **(B)** A scatter plot revealing the expression discrepancies of genes in two groups. The values of x- and y-axes are the normalized signal values of samples in two groups. Red, the significantly upregulated genes; green, the markedly downregulated genes. **(C)** A volcano plot demonstrating the DEGs in two groups with *P* value ≤ 0.05 and |log_2_(fold change)| ≥ 1 as the threshold. The red dots represent 37 upregulated genes and the green dots show 23 downregulated genes in the N315 group compared with their expression levels in the N315Δ*SprC* group. Each dot represents one gene. **(D)** A heat map of DEGs. T1-T3 are wild-type N315 samples and T4-T6 are N315Δ*SprC* samples. Euclidean distances between samples are used, and each sample value is chosen to plot the DEseq2 rlog-transformed value. Red, upregulated genes; blue, downregulated genes. Each line represents one gene. DEGs, differentially expressed genes.

To identify DEGs in *S*. *aureus* N315 following *sprC* knockout, we utilized DESeq2 program to yield *S*. *aureus* gene expression profiles. The program identified a total of 2497 genes expressed, among which 60 (2.4%) were differentially regulated, including 37 (1.5%) significantly up-regulated and 23 (0.9%) distinctly down-regulated DEGs (**Figure 1A**). The scatter plot revealed the expression discrepancies of genes between the wild-type strain and the knockout strain (**Figure 1B**). In addition, a volcano plot was created to picture the DEGs intuitively between both groups (**Figure 1C**). The heat map visualized the global expression changes of 60 DEGs between N315 and N315Δ*sprC* (**Figure 1D**). From the heat map, we apparently recognized that the expressions of the genes encoding leucocidin ED (SAOUHSC_01955 and SAOUHSC_01954) were markedly up-regulated in N315Δ*sprC*, which were associated with impetigo, antibiotic-associated diarrhea, and bloodstream infections (Yang et al., 2020). Detailed information about each DEG is available in **Supplementary Table 2**. The representatives of the significant DEGs involved in metabolism and virulence are listed in **Table 2**.

**Table 2:** The representatives of the significant differentially expression genes (DEGs) concerning metabolism and virulence in *S. aureus sprC* mutant compared with wild-type strain.

Gene ID	Gene name	Log_2_(fold change)	Function/Description	Definition
SAOUHSC_00707	*fruB*	1.14	Fructose and mannose metabolism	fructose 1-phosphate kinase
SAOUHSC_00871	*dltc*	1.31	D-Alanine metabolism, two-component system, cationic antimicrobial peptide (CAMP) resistance, *Staphylococcus aureus* infection	D-alanine–poly (phosphoribitol) ligase subunit 2
SAOUHSC_01009	*purK*	1.26	Biosynthesis of secondary metabolites, biosynthesis of antibiotics, purine metabolism	Phosphoribosylaminoimidazole carboxylase ATPase subunit
SAOUHSC_01010	*purC*	1.32	Biosynthesis of secondary metabolites, biosynthesis of antibiotics, purine metabolism	Phosphoribosylaminoimidazole-succinocarboxamide synthase
SAOUHSC_01012	*purQ*	1.27	Biosynthesis of secondary metabolites, biosynthesis of antibiotics, purine metabolism	Phosphoribosylformylglycinamidine synthase I
SAOUHSC_01013	*purL*	1.55	Biosynthesis of secondary metabolites, biosynthesis of antibiotics, purine metabolism	Phosphoribosylformylglycinamidine synthase II
SAOUHSC_01014	*purF*	1.25	Biosynthesis of secondary metabolites, biosynthesis of antibiotics, purine metabolism, Alanine, aspartate and glutamate metabolism	Amidophosphoribosyltransferase
SAOUHSC_01015	*purM*	1.49	Biosynthesis of secondary metabolites, biosynthesis of antibiotics, purine metabolism	Phosphoribosylaminoimidazole synthetase
SAOUHSC_01016	*purN*	1.56	Biosynthesis of secondary metabolites, biosynthesis of antibiotics, purine metabolism, one carbon pool by folate	Phosphoribosylglycinamide formyltransferase
SAOUHSC_01017	*purH*	1.75	Biosynthesis of secondary metabolites, biosynthesis of antibiotics, purine metabolism, one carbon pool by folate	Bifunctional Phosphoribosylaminoimidazolecarboxamide formyltransferase/IMP cyclohydrolase
SAOUHSC_01018	*purD*	1.14	Biosynthesis of secondary metabolites, biosynthesis of antibiotics, purine metabolism	Phosphoribosylamine–glycine ligase
SAOUHSC_01452	*ald*	1.15	Taurine and hypotaurine metabolism, metabolic pathways, alanine, aspartate and glutamate metabolism	Alanine dehydrogenase
SAOUHSC_01646	*glcK*	1.01	Glycolysis/gluconeogenesis, starch and sucrose metabolism, streptomycin biosynthesis, amino sugar and nucleotide sugar metabolism, metabolic pathways, biosynthesis of secondary metabolites, biosynthesis of antibiotics, microbial metabolism in diverse environments, carbon metabolism, galactose metabolism	Glucokinase
SAOUHSC_01807	*pfk*	1.14	Glycolysis/gluconeogenesis, methane metabolism, pentose phosphate pathway, biosynthesis of amino acids, RNA degradation, metabolic pathways, biosynthesis of secondary metabolites, biosynthesis of antibiotics, fructose and mannose metabolism, microbial metabolism in diverse environments, carbon metabolism, galactose metabolism	6-phosphofructokinase
SAOUHSC_02329	*thiM*	1.02	Metabolic pathways, thiamine metabolism	Hydroxyethylthiazole kinase
SAOUHSC_02402	*mtlA*	1.38	Phosphotransferase system (PTS), fructose and mannose metabolism	PTS system mannitol-specific transporter subunit IIA
SAOUHSC_02403	*mtlD*	1.05	Fructose and mannose metabolism	Mannitol-1-phosphate 5-dehydrogenase
SAOUHSC_02811	*SA2297*	1.22	Purine metabolism	Putative GTP pyrophosphokinase
SAOUHSC_00217	*SA0239*	-1.37	Sorbitol dehydrogenase; alcohol dehydrogenase	L-iditol 2-dehydrogenase
SAOUHSC_01954	*lukD*	-5.15	Leukocidin D	Leukotoxin LukD
SAOUHSC_01955	*lukE*	-4.98	Leukocidin E	Leukotoxin LukE
SAOUHSC_02411		-2.03		Hypothetical protein
SAOUHSC_02412		-1.87		Hypothetical protein
SAOUHSC_02721		-1.56		Hypothetical protein

The representatives of the significant DEGs concerning metabolism and virulence. The definitions and gene names of these DEGs are also represent in the table to show their functions in metabolism and virulence. The DEGs were identified using DESeq2 v 1.10.1 package in R language (|log_2_(fold change)| ≥1, false discovery rate (FDR) ≤ 0.05).

### Go Functional Enrichment Analysis

**Figure 2:**
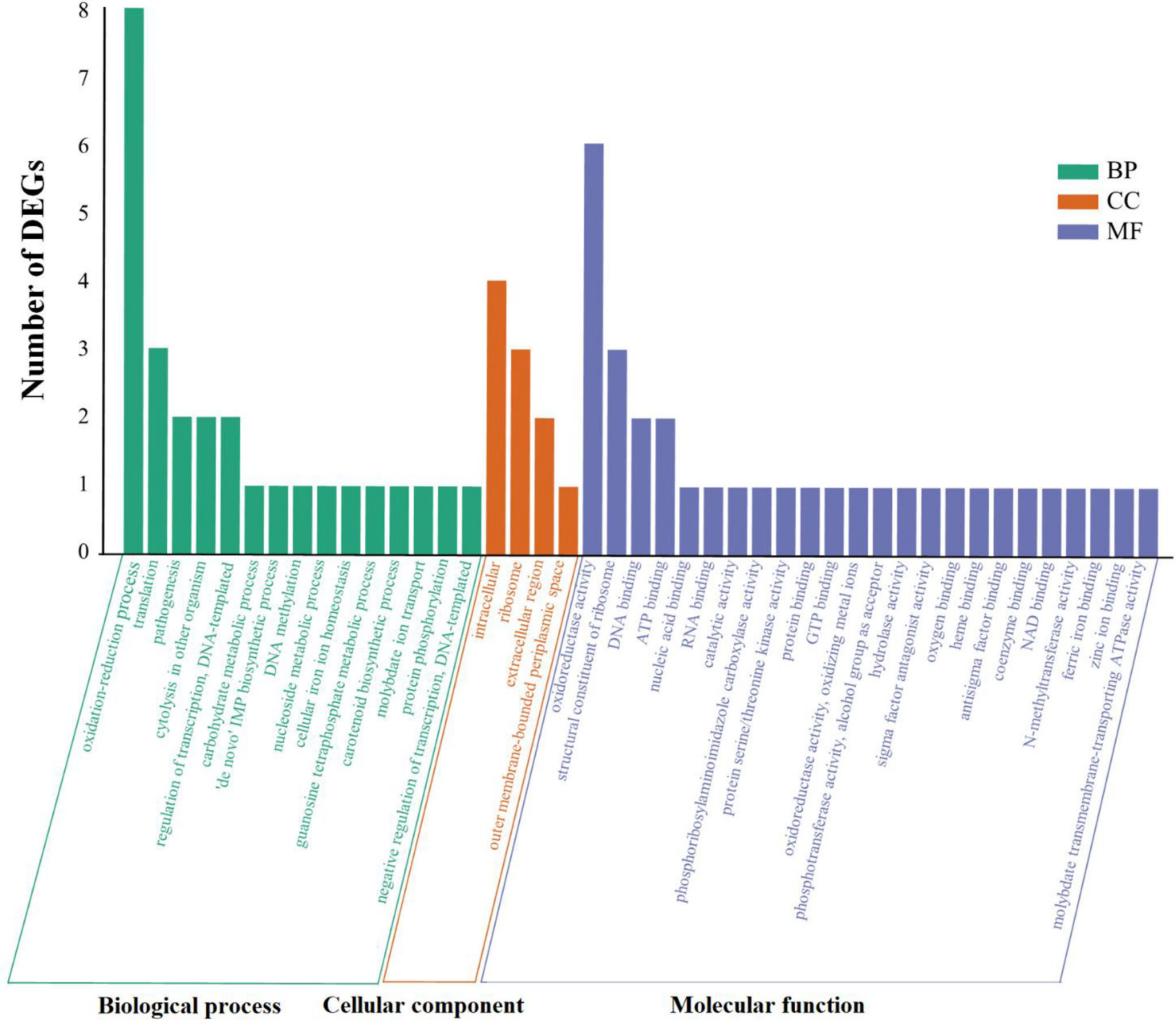
All the GO terms enriched among the DEGs. Go databases were used to conduct GO functional annotation on the DEGs by R package GOseq v 1.18. All annotated determinants (x-axis) are divided into 3 GO domains: biological process (15 terms), molecular function (4 terms) and cellular component (24 terms). The y-axis suggests the number of DEGs. Green histogram stands for the biological process (BP), the orange histogram indicates cellular component (CC), and the purple histogram represents molecular function (MF). Differential expression profiles are acquired from DEGs in the wild-type and mutant strains, disclosing the impact of SprC on *S*. *aureus* metabolism, physiology and pathogenesis. GO, Gene Ontology; DEGs, differentially expressed genes.

To further understand the possible effects of SprC on DEGs, GO functional enrichment analysis was conducted. Our results displayed that the significantly enriched annotations were related to three GO domains: biological process [15 terms, such as oxidation-reduction process (8 genes), translation (3 genes), regulation of transcription (2 genes) and pathogenesis (2 genes)], molecular function [24 terms, most involved in catalytic activity (13 genes) and binding (15 genes)], and cellular component [4 terms, most belonging to intracellular (4 genes) and ribosomal components (3 genes)] (**Figure 2**). Discrepant transcription profiles acquired from the DEGs in wild-type and knockout strains indicated a potential effect of SprC on *S. aureus* metabolism and pathogenicity.

### KEGG Pathway Enrichment Analysis

**Figure 3:**
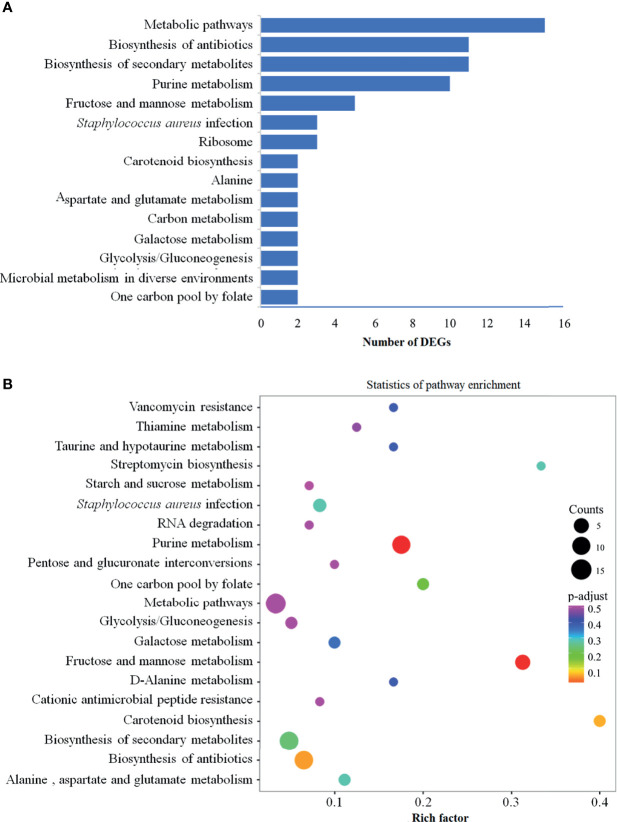
KEGG pathway enrichment analysis of DEGs. KEGG pathway enrichment analysis was conducted based on the KEGG database (R package GOseq v 1.18). **(A)** Top 15 enriched KEGG pathways among DEGs. These 15 pathways are arranged in the order of number of DEGs, and the pathways enriched the most DEGs are metabolic pathways, followed by biosynthesis of antibiotics, biosynthesis of secondary metabolites and purine metabolism. **(B)** Visualization of the 20 most significant KEGG enrichment pathway. The 20 most significant pathways were selected by combining three factors: enrichment factor (x-axis), *P* value (color of the dots) and number of genes enriched (size of the dots). KEGG, Kyoto Encyclopedia of Genes and Genomes; DEGs, differentially expressed genes.

In order to further identify the functions of DEGs, all DEGs were mapped to KEGG database for the KEGG pathway enrichment analysis. Totally, 32 pathways were enriched, and the detailed information was presented in **Supplementary Table 2**. From **Supplementary Table 2**, we observed that some determinants were involved in several biological activities, and some were limited to a single pathway. The top four clearly enriched pathways, namely metabolic pathways, biosynthesis of antibiotics, biosynthesis of secondary metabolites and purine metabolism were shown in **Figure 3A**. In addition, data represented in **Figure 3A** reflected that there were 15 genes mapped to metabolic pathways, 11 genes involved in the pathways of biosynthesis of antibiotics, 11 genes mapped to biosynthesis of secondary metabolites, and 10 genes related to purine metabolism pathway. Besides, the pathways involving fructose and mannose metabolism (5 genes), *Staphylococcus aureus* infection and ribosome (3 genes each) were also remarkedly enriched (**Figure 3A**). A bubble chart (**Figure 3B**) generated with factors of enrichment factor, *P* value and numbers of DEGs revealed the 20 most significant pathways, among which the above mainly pathways were included.

### Validation of Differential Expression Genes

**Figure 4:**
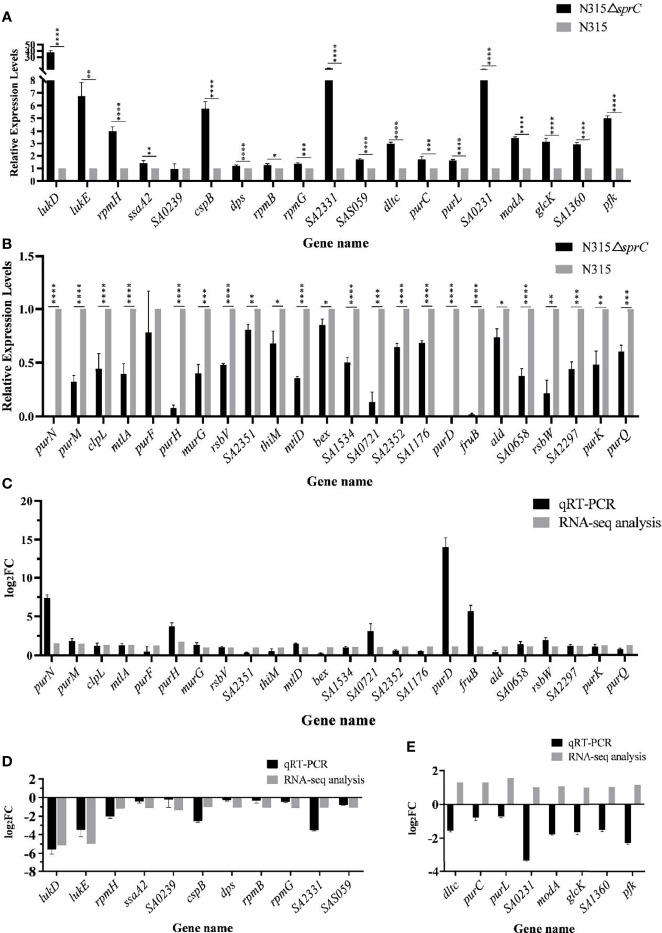
qRT-PCR analysis for validation of expression levels of 43 DEGs with defined function between N315 and N315Δ*SprC* strains. **(A)** 19 DEGs were up-regulated after the *sprC* knockout analyzed by qRT-PCR. **(B)** 24 DEGs were down-regulated after the *sprC* deletion. Black bar, strain N315Δ*sprC*; gray bar, strain N315 *P < 0.05, **P < 0.01, ***P < 0.001, ****P < 0.0001. The consistence of the results between RNA-seq and qRT-PCR analyzed with log_2_(Fold change) (log2FC) (y-axis) was shown in **(C–E)**. **(C, D)** 24 up-regulated and 11 down-regulated genes by SprC were validated consistent between both methods. **(E)** The expressions of 8 genes detected by qRT-PCR were validated opposite to those of RNA-seq. Black bar, qRT-PCR; gray bar, RNA-seq; qRT-PCR, quantitative real-time polymerase chain reaction; DEGs, differentially expressed genes.

To confirm the accuracy of gene expression detected by RNA-seq, the DEGs with a defined function including 32 up-regulated genes and 11 down-regulated genes were reanalyzed using qRT-PCR. As indicated in **Figure 4**, the mRNA levels of 35 of 43 DEGs (81.4%) assessed by qRT-PCR were congruent with the results of RNA-seq (a consistent trend for two genes with no significant difference). However, opposite results were shown for the remaining 8 genes (*dltc*, *purC*, *purL*, *SA0231*, *modA*, *glcK*, *SA1360* and *pfk*) (**Figure 4E**). Although there were several contradictory results obtained by both methods, the data from RNA-seq were generally reliable.

**Figure 5:**
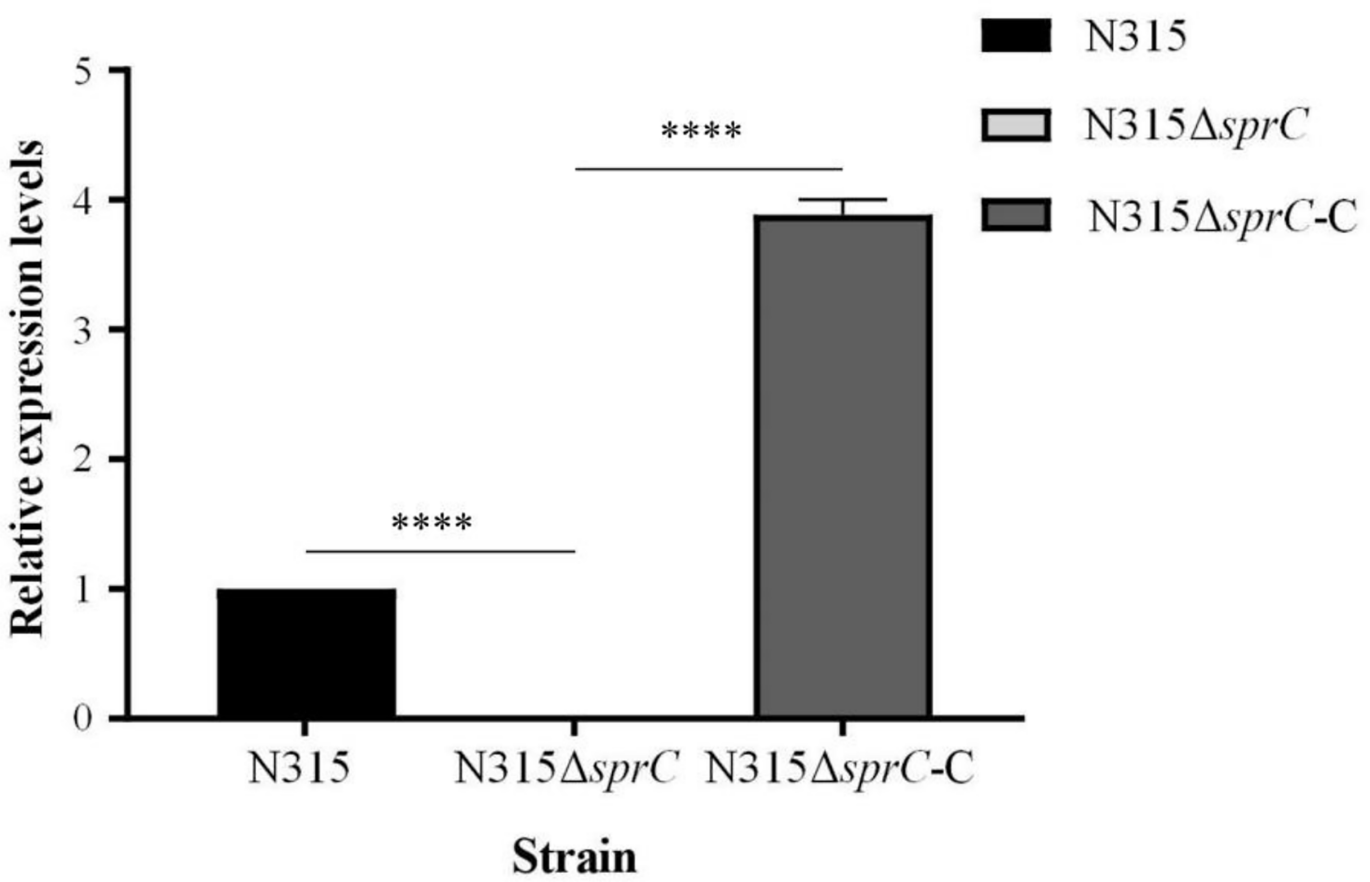
Relative expression of *sprC* in N315 wild-type, knockout and complementation strains. The knockout strain (N315Δ*sprC*) was constructed *via* temperature-sensitive plasmid pKOR1 by homologous recombination. The complementation strain (N315Δ*sprC*-C) was constructed by transferring recombinant plasmid pOS1-*sprC* into strain N315Δ*sprC via* electroporation. The levels of expression of *sprC* in the three strains were detected by qRT-PCR. The *sprC* is barely expressed in the knock out strain N315Δ*sprC*, and restored to the level of wild-type strain in the complementation strain N315Δ*sprC*-C. black bar, N315, the wild-type strain; gray bar, N315Δ*sprC*, the knock out strain; deep gray bar, N315Δ*sprC*-C, the complementation strain. ****P < 0.0001.

In order to further verify the influence of SprC on gene expression, the complementation strain N315Δ*sprC*-C was constructed, screened and verified (**Supplementary Figure 1** and **Supplementary Sequencing Data**). The result of qRT-PCR showed that the expression level of *sprC* in the complementation strain was returned to that in the wild-type N315 strain (**Figure 5**). Further analysis discovered that the mRNA levels of the 10 randomly selected DEGs in the complementation strain were also restored to those in the wild-type strain (**Supplementary Figure 2**).

### Prediction of Putative Targets of SprC

We predicted the binding of SprC with the mRNA transcribed from the DEGs, except for genes encoding hypothetical proteins. The Mfes of SprC:target mRNA are listed in **Table 3**. According to Akhtar et al. (2019), low level of Mfe means that an RNA-RNA duplex is thermodynamically stable and it is more difficult to break up the base-paring between RNA strands. A tight RNA-RNA combination is more likely to be viewed as a real target (Akhtar et al., 2019). As shown in **Table 3**, the predicted Mfes of SprC:mRNA were exceptionally low (-95.5 to-54.4), signaling that SprC and the mRNAs predicted could bind spontaneously with a good affinity. The predicted diagram of the secondary structure of the binding between SprC and mRNA is given in **Supplementary Figure 3**.

**Table 3:** Molecular free energy (Mfe) obtained by SprC:mRNA of DEGs prediction.

DEG ID	DEG name	Mfe(kcal/mol)		DEG ID	DEG name	Mfe(kcal/mol)
SAOUHSC_01018	*purD*	-95.5		SAOUHSC_01626	*SA1360*	-82.5
SAOUHSC_01009	*purK*	-94.8		SAOUHSC_01821	*SA1534*	-82.5
SAOUHSC_00707	*fruB*	-93.5		SAOUHSC_01954	*lukD*	-81.7
SAOUHSC_01013	*purL*	-92.9		SAOUHSC_01668	*bex*	-81.6
SAOUHSC_02402	*mtlA*	-92.6		SAOUHSC_02549	*modA*	-81.5
SAOUHSC_00204	*SA0231*	-90.1		SAOUHSC_00712	*SA0658*	-80.7
SAOUHSC_01012	*purQ*	-88.8		SAOUHSC_02299	*rsbW*	-80
SAOUHSC_00217	*SA0239*	-88.5		SAOUHSC_02381	*dps*	-78.2
SAOUHSC_02862	*clpl*	-87.9		SAOUHSC_01646	*glcK*	-75.8
SAOUHSC_01017	*purH*	-86.5		SAOUHSC_01010	*purC*	-73.9
SAOUHSC_02571	*ssaa2*	-85.7		SAOUHSC_02811	*SA2297*	-73.8
SAOUHSC_01807	*pfk*	-85.6		SAOUHSC_02882	*SA2352*	-72.1
SAOUHSC_02403	*mtlD*	-85.3		SAOUHSC_01191	*rpmB*	-69.9
SAOUHSC_01014	*purF*	-85.3		SAOUHSC_02300	*rsbV*	-67.3
SAOUHSC_02881	*SA2351*	-85.3		SAOUHSC_00871	*dltc*	-66.1
SAOUHSC_01452	*ald*	-84.9		SAOUHSC_03045	*cspB*	-65.7
SAOUHSC_00788	*SA0721*	-84.4		SAOUHSC_03055	*rpmH*	-61
SAOUHSC_01955	*lukE*	-83.7		SAOUHSC_02853	*SA2331*	-59.8
SAOUHSC_01424	*murG*	-83.6		SAOUHSC_01336	*SA1176*	-54.4
SAOUHSC_02329	*thiM*	-82.9				

All the mRNAs transcribed by DEGs with defined functions were predicted the ability to bind SprC by a standalone algorithm RNAhybrid on Bielefeld Bioinformatics Service website (https://bibiserv.cebitec.uni-bielefeld.de/). Molecular free energies (Mfes) are listed from the smallest to the largest (-95.5 to-54.4). The low values of Mfes indicated a good affinity of sprC and mRNAs binding together. DEG, differentially expressed gene.

### Differential Metabolites Analysis

**Figure 6:**
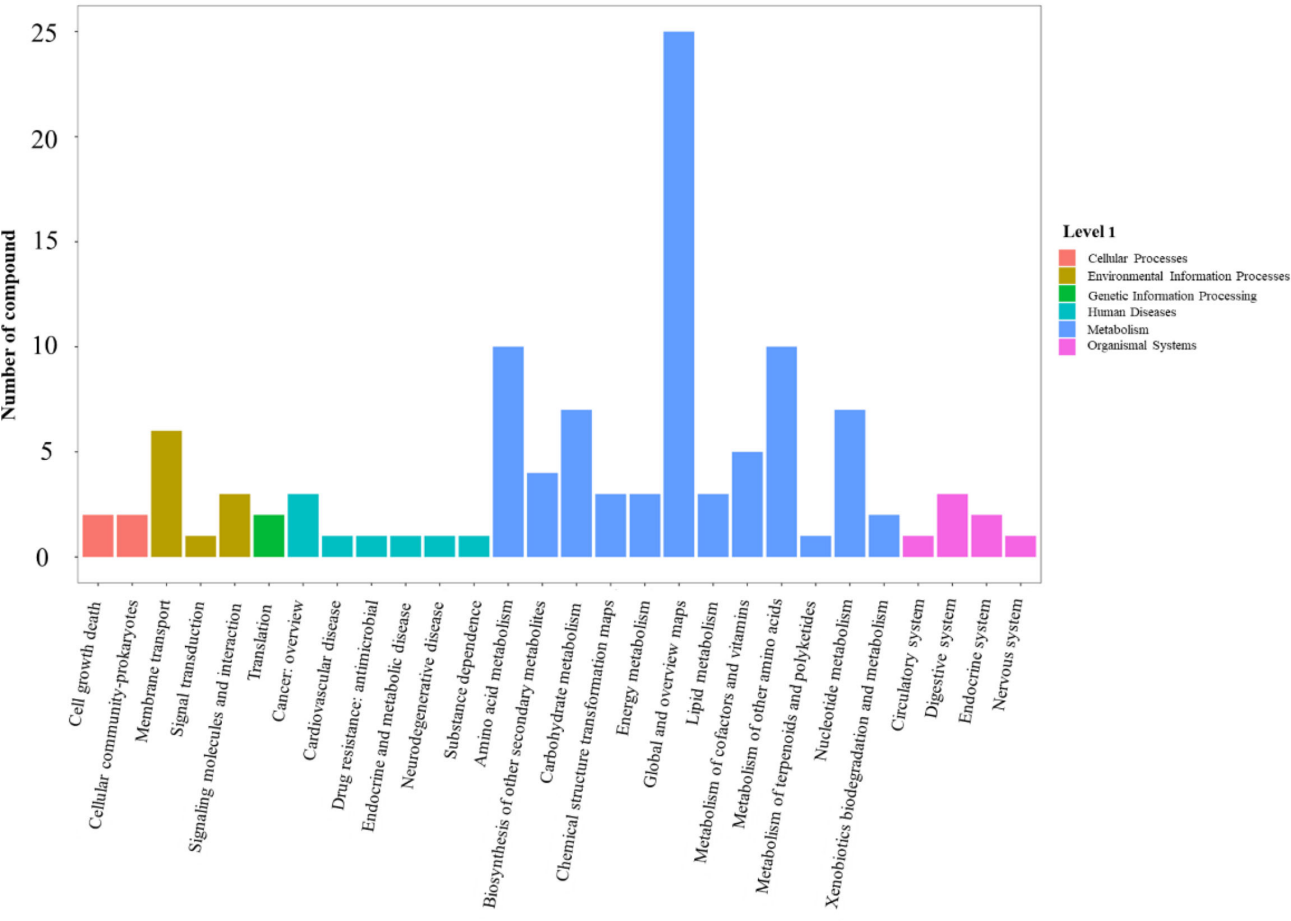
Histogram of ions and metabolites annotated to KEGG pathways. The fold changes were used to identify differential metabolites based on partial least squares method-discriminant analysis (PLS-DA) and variable importance in projection (VIP) value. Pathways with differential metabolite were enriched using KEGG pathway database. X-axis shows the pathways of level 2 derived from pathways of level 1 (cellular processes, environmental information processing, genetic information processing, human diseases, metabolism and organismal system) in KEGG database. Y-axis shows the number of compounds enriched in the pathway. The significantly enriched pathways (such as global and overview maps, amino acid metabolism and metabolism of other amino acid) are pathways from metabolism (blue bar). KEGG, Kyoto Encyclopedia of Genes and Genomes.

With the raw mass spectrometry data imported from the LC-MS/MS acquisition, 7123 metabolites were identified. Compared with the wild-type strain N315, 256 differential ions (3.6%) were screened based on the conditions of ratio ≥ 2 or ≤ 1/2, *P* value ≤ 0.05 and VIP ≥ 1 in *sprC* deletion mutant strain (N315Δ*sprC*), including 187 (2.6%) up-regulated ions and 69 (1.0%) down-regulated ions. All the differentially regulated metabolites are available in **Supplementary Table 3**. KEGG pathways analysis indicated that these differential metabolites were enriched in various KEGG pathways of level 1, including cellular processes, environmental information processing, genetic information processing, human diseases, metabolism and organismal system, and the pathways of metabolism enriched the maximum amount of differential metabolites (**Figure 6**). Among the level 2 pathways of metabolism, we observed that global and overview maps enriched the most differential metabolites (25 metabolites), and amino acid metabolism and metabolism of other amino acid each enriched 10 metabolites (**Figure 6**). Specific pathway enrichment derived from level 2 pathways using the compounds in the KEGG database was presented as a bubble chart in **Supplementary Figure 4**, in which 77 specific pathways were identified and many, such as metabolic pathways (the most significantly enriched pathways, including 28 metabolites), biosynthesis of secondary metabolites and purine metabolism, were consistent with those enriched in the RNA-seq data.

## Discussion

*S. aureus* possesses complex regulatory circuits, which enable the survival and proliferation of the pathogen after infecting the host. sRNAs, also known as noncoding RNAs, act primarily as a moderator of translation and mRNA stability (Jørgensen et al., 2020). The majority of sRNAs are predominantly encoded in the core genome, with a few localized in mobile elements, pathogenicity islands or plasmids (Pichon and Felden, 2005). Recent studies have enriched the regulatory mechanisms employed by sRNAs in *S. aureus*, among which the regulation function based on base pairing is the most commonly demonstrated (Caldelari et al., 2013; Jørgensen et al., 2020). In view of the significance of sRNAs, we constructed the isogenic *sprC* deletion mutant strain of *S. aureus* N315 in order to uncover the impact of this sRNA on bacterial gene expression. RNA-seq is a crucial tool in the study of transcriptomics (Stark et al., 2019), so this technology was used by us to acquire the differential gene expression data, which were further analyzed to reveal the functions related to these genes by GO and KEGG pathway analysis.

The discoveries that SprC attenuates the virulence, leukocytic phagocytosis of *S. aureus* and anti-oxidative stress have been confirmed (Le Pabic et al., 2015). Proteomics analysis showed that the proteins modulated by SprC are mainly connected to metabolic and cellular processes, biological regulation and catalytic activity (Zhao et al., 2017). In this study, the transcriptome data also exhibited the regulatory functions of SprC on metabolism, enzymatic activity and virulence of *S. aureus*. In line with the above two omics analysis, metabolomics revealed that the differential metabolites affected by SprC were also mainly related to metabolism, environmental information processing and cellular processes.

It is well known that biofilm is an essential part contributing to the virulence of *S. aureus* (Schilcher and Horswill, 2020). Moreover, the formation of biofilm has much to do with the surface compositions of bacteria (Moormeier and Bayles, 2017). D-alanyl carrier protein plays an important role in the biosynthesis of lipoteichoic acid (LTA), which is able to modulate the properties of the cell wall of gram-positive bacteria and inhibit biofilm formation of *S. aureus* (Rajagopal and Walker, 2017; Ahn et al., 2018). Our findings showed that *dltC* gene encoding D-alanyl carrier protein was remarkably up-regulated by SprC. Therefore, we speculated that SprC could exert an influence on the inhibition of the biofilm formation of *S. aureus*. Based on the transcriptome and metabolome analysis, we found that many genes and metabolites were affected by SprC to varying degrees, especially those related to ABC transporters. ABC transporter is closely associated with the formation of *S. aureus* biofilm and is also involved in drug resistance of bacteria (McCarthy et al., 2015). According to the DEGs data, we found that some classical virulence or regulation factors, such as *lukD* (SAOUHSC_01954) and *lukE* (SAOUHSC_01955), were significantly down-regulated in N315 strain compared with N315Δ*sprC* strain. LukED belongs to the family of synergohymenotropic toxins that can harm host cells and play a key role in modulating the progress of *S. aureus* infection (Ahn et al., 2018; Yang et al., 2020). *sprC* gene is located next to *lukE/D*, and they have good spontaneous binding affinity according to our SprC:mRNA prediction results. Therefore, it is rational to infer that SprC could modulate the expression of LukED by directly binding to its mRNA. However, this speculation, which was also applied to other identified DEGs, needs to be further validated.

The determinants involved in sugar metabolism were also found in the DEGs. Sugars are the main source of carbohydrates used by bacteria. Bacteria can bind several sugars in the cytoplasm and utilize these sugars to produce ATP through glycolysis or generate polysaccharides by polymerization (Komatsuzawa et al., 2004). Study showed that there are tight links between the regulation of sugar metabolism and the virulence of *S. aureus* (Seidl et al., 2008). Carbohydrate phosphotransferase system (PTS), a major carbohydrate active transport system, is found only in bacteria. This system can catalyze the phosphorylation of sugars, concomitantly transport the sugars across the cell membrane, and interfere with bacterial virulence (Postma et al., 1993; Deutscher et al., 2006). This study showed that SprC could impact PTS by regulating *mtlA* (SAOUHSC_02402) encoding one PTS enzyme. ATP-dependent 6-phosphofructokinase encoded by SAOUHSC_01807 (*pfkA*) is the key enzyme in the first committing step of glycolysis (Tian et al., 2018). However, whether SprC has the function of regulating the expression of this enzyme remains to be confirmed, because in this study there were inconclusive results on the expression of *pfkA* (**Figure 4**). Additionally, genes SAOUHSC_00707, SAOUHSC_01807, SAOUHSC_02402 and SAOUHSC_02403 up-regulated by SprC, as shown in **Supplementary Table 2**, might result in an increased expressions of β-D-fructose 1, 6-bisphosphate and enhanced glycolysis (Deng et al., 2014; Gong et al., 2017; Li et al., 2018; Chen et al., 2019). Luo et al. demonstrated that the growth rate of *S. aureus* was reduced under high glucose conditions due to impairment of the pentaglycine bridge contributing to the bacterial cell wall structure (Luo et al., 2020). Therefore, SprC might provide one approach to maintain cell stability by increasing the expression of genes related to upgraded glycolysis. Metabolomics results also revealed some enriched pathways in glucose and carbon metabolism, such as C5-branched dibasic acid metabolism. Previous studies suggested that multiple factors in amino acid metabolism moonlight as regulators in the expressions of genes regulating bacterial virulence (Lomas-Lopez et al., 2007; Frank et al., 2021). Our findings also showed that amino acid metabolism was significantly enriched according to KEGG analysis of metabolome and transcriptome data. As a result, SprC could influence the virulence of *S*. *aureus* by regulating nutritional conditions, such as amino acid metabolism.

Purines, required for the synthesis of nucleic acids and adenosine triphosphate (ATP), are essential to the intracellular growth of *S. aureus* (Goncheva et al., 2020). The production and metabolism of purines in cells is a very complex process in which a variety of enzymes and physiological conditions are involved to maintain cellular homeostasis (Maiuolo et al., 2016). As presented in **Supplementary Table 2**, the expressions of ten determinants (SAOUHSC_01009, 01010, 01012-01018 and 02811) associated with the “purine metabolism” were up-regulated by SprC. These genes (except SAOUHSC_02811) encode the catalytic enzymes in the hypoxanthine nucleotide (IMP) biosynthesis *via de novo* pathway that would consume ATP (Schultheisz et al., 2008). The decline in ATP may induce resistance to antibiotics (Conlon et al., 2016). Furthermore, previous investigations showed that the transcription levels of all genes in the purine (*pur*) operon are much higher in vancomycin-resistant *S. aureus* strains than in vancomycin-sensitive strains (Mongodin et al., 2003; Fox et al., 2007). Based on the metabolomics data, differential metabolites were also enriched in energy metabolism and purine metabolism pathways. For example, adenosine, a component of purine metabolism, was down-regulated in N315Δ*sprC* strain. In addition, some metabolites related to pyrimidine metabolism and intermediate products of pyrimidine metabolism including uridine and thymidine were also decreased in N315Δ*sprC* strain. Evidence of vancomycin resistance variability due to gene deletion was also provided by our metabolomics data (**Figure 3B**). Together, our data suggested that SprC might also impact the properties of *S. aureus* by regulating nucleotide metabolism.

Numerous early studies elucidated the complex relationships between metabolism and virulence regulation in *S. aureus* and revealed that alterations in virulence may be induced by changes in metabolism (Somerville and Proctor, 2009; Balasubramanian et al., 2017; Richardson, 2019). In this study, the observation of the effects of SprC on the expression of *S. aureus* metabolism-related genes might provide an interpretation for the ability of SprC to reduce the virulence of *S. aureus*. And our results of both metabolomics and complementation strain construction analysis also demonstrated the correctness of this conclusion.

In conclusion, 60 DEGs were discovered to be affected by SprC. Analyzed by GO annotation and KEGG pathway, these influenced determinants were mainly associated with metabolic processes (such as sugar and purine metabolism), catalytic functions, substrate binding and *S. aureus* infection. These findings were similar to our previous proteomic data on the effects of SprC (Zhao et al., 2017). Additionally, in this study that SprC affected the metabolism of *S. aureus* further supported the viewpoint of SprC regulating the virulence of *S. aureus*. Based on all these findings, SprC, a multifaceted RNA, might be identified as a metabolic signature and a potential target, which can be employed for drug discovery and development for the adjuvant treatment of drug-resistant bacterial infections. Nevertheless, there were several limitations in our study. For the first, no experiments were performed to verify whether sRNA SprC directly or indirectly regulated the expression of the identified DEGs. Second, we did not conduct further research to elucidate the mechanisms underlying the regulatory function of SprC.

## Data availability statement

The datasets presented in this study can be found in online repositories. The names of the repository/repositories and accession number(s) can be found in the article/**Supplementary Material**.

## Author Contributions

JZ and HZ carried out the experiments and wrote the manuscript; they contributed equally to this work and shared first authorship. HY, CH, WS and ZC analyzed the data and interpreted the results. QL designed the experiments and corrected the manuscript. All authors contributed to the article and approved the submitted version.

## Funding

This work was supported by grants from the National Natural Science Foundation of China (No. 81772247 and No. 81371872) and Natural Science Foundation, Science and Technology Commission of Shanghai (20ZR1444800).

## Conflict of Interest

The authors declare that the research was conducted in the absence of any commercial or financial relationships that could be construed as a potential conflict of interest.

## Publisher’s Note

All claims expressed in this article are solely those of the authors and do not necessarily represent those of their affiliated organizations, or those of the publisher, the editors and the reviewers. Any product that may be evaluated in this article, or claim that may be made by its manufacturer, is not guaranteed or endorsed by the publisher.

## Supplementary Material

Supplementary Figure 1 Agarose gel electrophoresis of double-digested plasmids. The recombinant plasmid was digested with endonucleases Nhel and BamHI, and 2 fragments of DNA, namely plasmid vector pOS1 and sprC, were produced. M1 and M2, DNA markers; lanes 1 and 2, results of enzymatic digestion of plasmids (pOS1-sprC) extracted from the complementation strain N315ΔsprC-C; lanes 3 and 4, results of enzymatic digestion of the recombinant plasmid pOS1-sprC.

Supplementary Figure 2 Relative expression of 10 randomly selected DEGs. 5 up-regulated and 5 down-regulated DEGs from RNA-seq data were evaluated in wild-type, knockout and complementation strains by qRT-PCR. The results showed that the expression of the selected DEGs detected by both methods was consistent, indicating the accuracy of the RNA-seq results. N315, wild-type strain; N315DsprC, the knock out strain; N315DsprC-C, the complementation strain; DEG, selected differentially expressed genes.

Supplementary Figure 3 Predicted secondary structure of the binding of mRNA transcribed by DEGs with defined function and SprC. The prediction was performed based on the bioinformatics tools on Bielefeld Bioinformatics Service (https://bibiserv.cebitec.uni-bielefeld.de/). The thermodynamic stability of SprC:mRNA duplex is calculated using a standalone algorithm RNAhybrid. Molecular free energy (Mfe) of < 0 indicates that SprC and mRNA can bind spontaneously with good affinity. Bases marked in red are SprC; green bases belong to DEGs.

Supplementary Figure 4 Bubble chart of metabolite specific pathway enrichment using the compounds in the KEGG database. All pathways were classified into 6 items, namely, cellular processes, environmental information processing, genetic information processing, human diseases, metabolism and organismal system. In the picture, a total of 52 pathways enriched in metabolism part are mainly related to nutrient metabolism. A total of 8 pathways involved in cellular processes (3 pathways) and environmental information processing (5 pathways) mainly focus on signaling systems. One Pathway from genetic information processing is centered on tRNA biosynthesis. 8 pathways from human diseases have a strong connection with drug resistance. The most significantly enrich pathway of 8 pathways in organismal systems is protein digestion and absorption.

Supplementary Table 1 The primer pairs used for the validation of DEGs expression.

Supplementary Table 2 DEGs in N315ΔsprC and N315 strains.

Supplementary Table 3 Statistical analysis of differential ions quantification data.

Supplementary Sequencing Data Partial sequence of pOS1-sprC.
