# Supplementary material for: Insights Into the Impact of Small RNA SprC on the Metabolism and Virulence of Staphylococcus aureus
Source: Front Cell Infect Microbiol. 2022 Feb 23;12:746746. doi: 10.3389/fcimb.2022.746746 (PMC8905650; doi:10.3389/fcimb.2022.746746)
Supplement: Supplementary Figure 1 — Agarose gel electrophoresis of double-digested plasmids. The recombinant plasmid was digested with endonucleases Nhel and BamHI, and 2 fragments of DNA, namely plasmid vector pOS1 and sprC, were produced. M1 and M2, DNA markers; lanes 1 and 2, results of enzymatic digestion of plasmids (pOS1-sprC) extracted from the complementation strain N315ΔsprC-C; lanes 3 and 4, results of enzymatic digestion of the recombinant plasmid pOS1-sprC. [file Image_1.pdf]

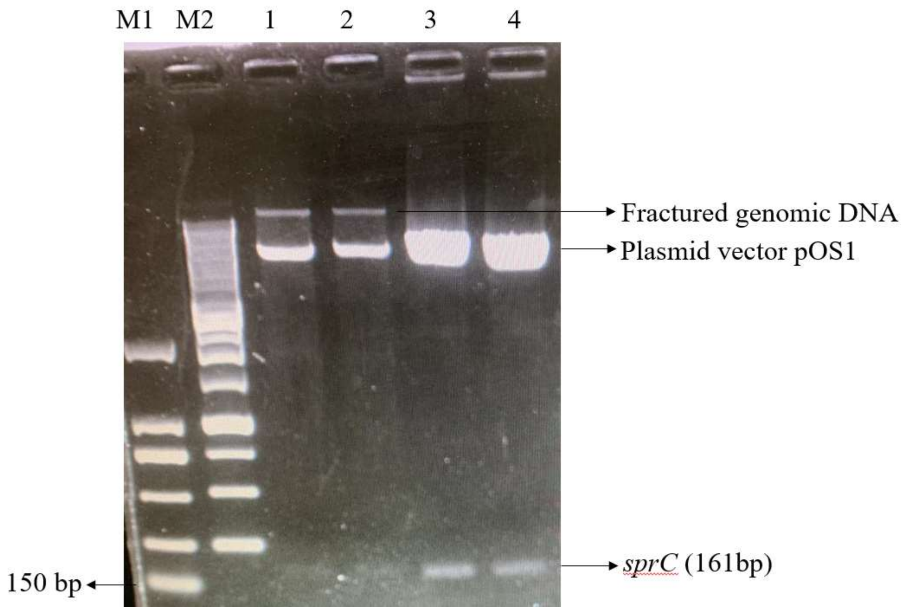

Supplementary Figure 1. Agarose gel electrophoresis of plasmids double digestion. The recombinant plasmid was digested with endonucleases NheI and BamHI, and produced 2 fragments of DNA, namely, plasmid vector pOS1 and *sprC*. M1 and M2, DNA markers; lanes 1 and 2, results of enzymatic digestion of plasmids (pOS1-*sprC*) extracted from the complementary strain N315Δ*sprC*-C; lanes 3 and 4, results of enzymatic digestion of the recombinant plasmid *sprC*-pOS1.
