# Supplementary figures and images for: Insights Into the Impact of Small RNA SprC on the Metabolism and Virulence of Staphylococcus aureus
Source: Front Cell Infect Microbiol. 2022 Feb 23;12:746746. doi: 10.3389/fcimb.2022.746746 (PMC8905650; doi:10.3389/fcimb.2022.746746)

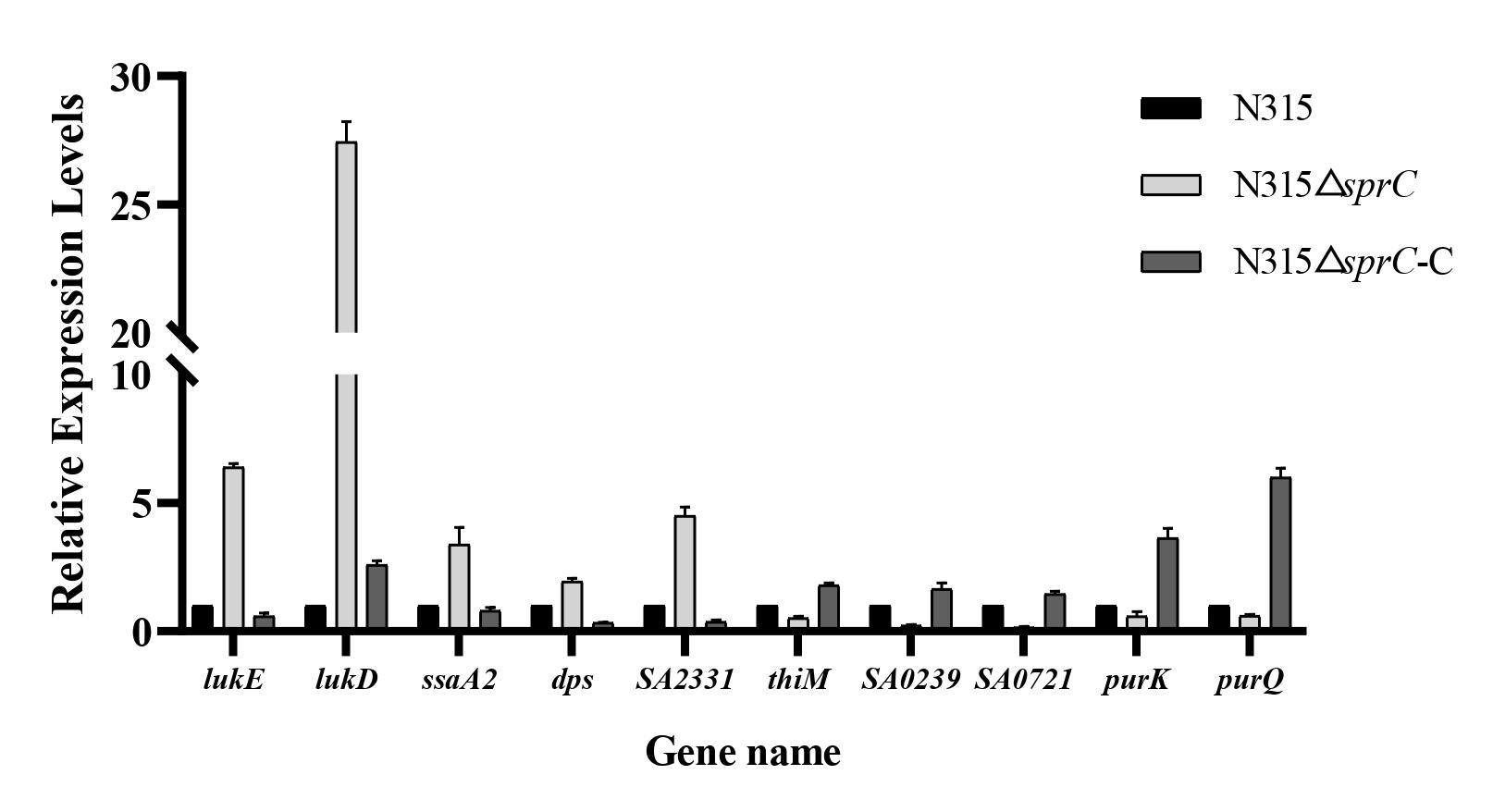

Supplement: Supplementary Figure 2 — Relative expression of 10 randomly selected DEGs. 5 up-regulated and 5 down-regulated DEGs from RNA-seq data were evaluated in wild-type, knockout and complementation strains by qRT-PCR. The results showed that the expression of the selected DEGs detected by both methods was consistent, indicating the accuracy of the RNA-seq results. N315, wild-type strain; N315DsprC, the knock out strain; N315DsprC-C, the complementation strain; DEG, selected differentially expressed genes. [file Image_2.jpg]

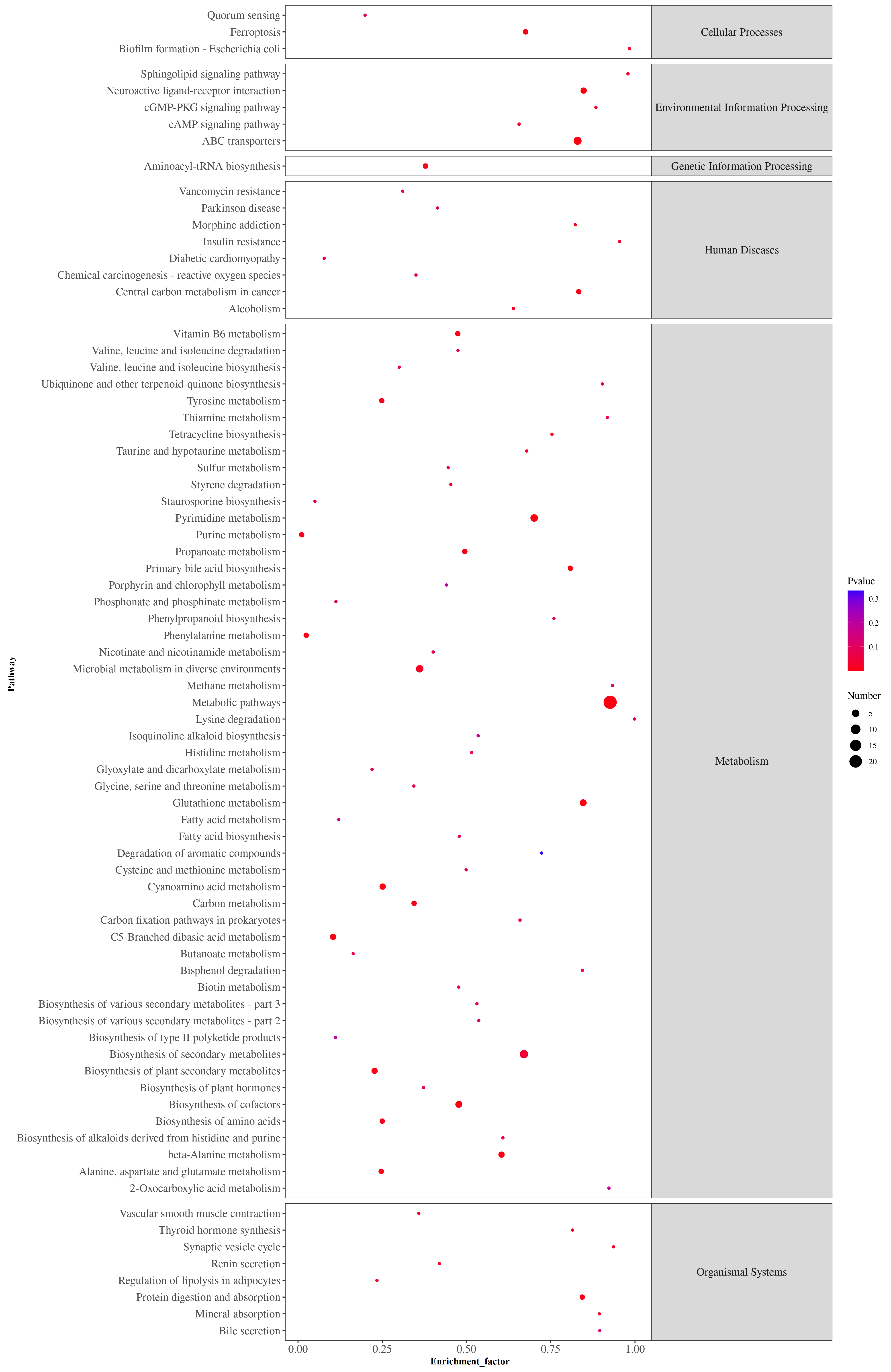

Supplement: Supplementary Figure 4 — Bubble chart of metabolite specific pathway enrichment using the compounds in the KEGG database. All pathways were classified into 6 items, namely, cellular processes, environmental information processing, genetic information processing, human diseases, metabolism and organismal system. In the picture, a total of 52 pathways enriched in metabolism part are mainly related to nutrient metabolism. A total of 8 pathways involved in cellular processes (3 pathways) and environmental information processing (5 pathways) mainly focus on signaling systems. One Pathway from genetic information processing is centered on tRNA biosynthesis. 8 pathways from human diseases have a strong connection with drug resistance. The most significantly enrich pathway of 8 pathways in organismal systems is protein digestion and absorption. [file Image_4.png]
