# Supplementary material for: Insights Into the Impact of Small RNA SprC on the Metabolism and Virulence of Staphylococcus aureus
Source: Front Cell Infect Microbiol. 2022 Feb 23;12:746746. doi: 10.3389/fcimb.2022.746746 (PMC8905650; doi:10.3389/fcimb.2022.746746)
Supplement: Supplementary Figure 3 — Predicted secondary structure of the binding of mRNA transcribed by DEGs with defined function and SprC. The prediction was performed based on the bioinformatics tools on Bielefeld Bioinformatics Service (https://bibiserv.cebitec.uni-bielefeld.de/). The thermodynamic stability of SprC:mRNA duplex is calculated using a standalone algorithm RNAhybrid. Molecular free energy (Mfe) of < 0 indicates that SprC and mRNA can bind spontaneously with good affinity. Bases marked in red are SprC; green bases belong to DEGs. [file Image_3.pdf]

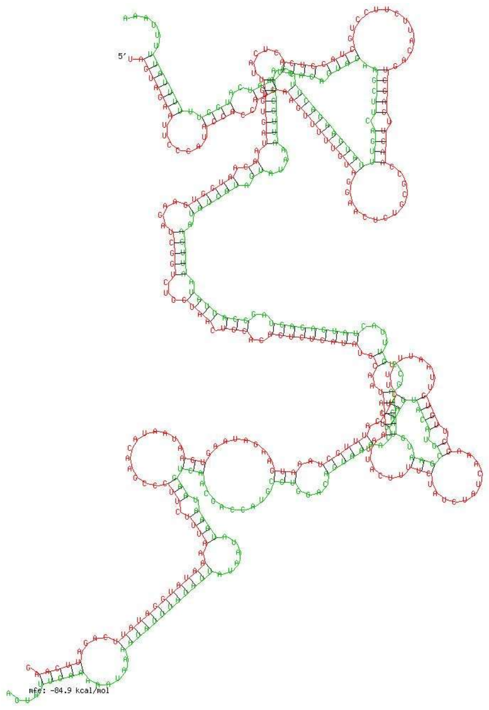


ald:SprC

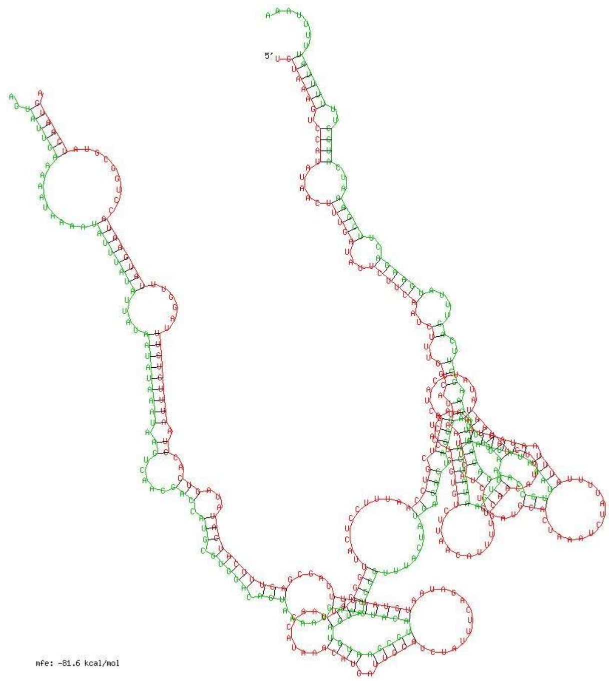


bex:SprC

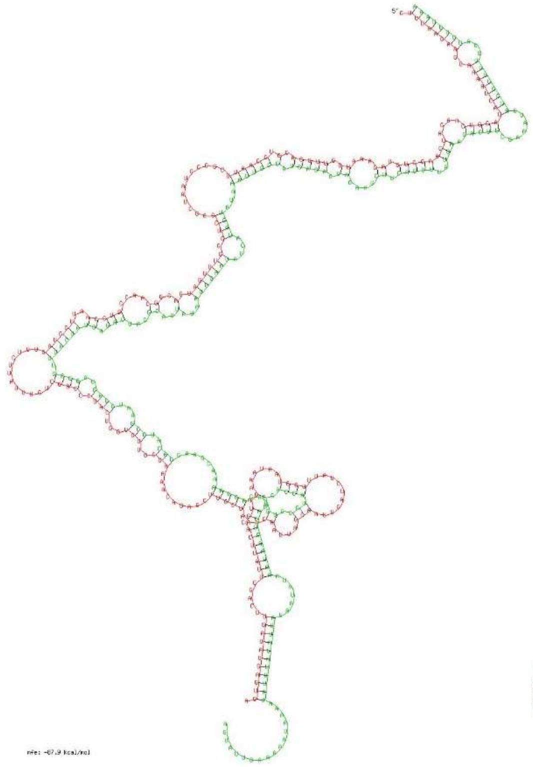


clpL:SprC

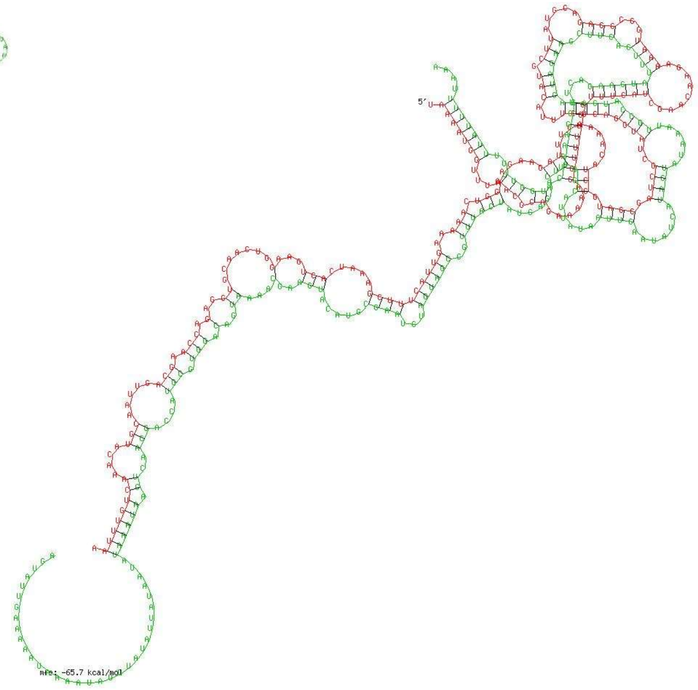


cspB:SprC

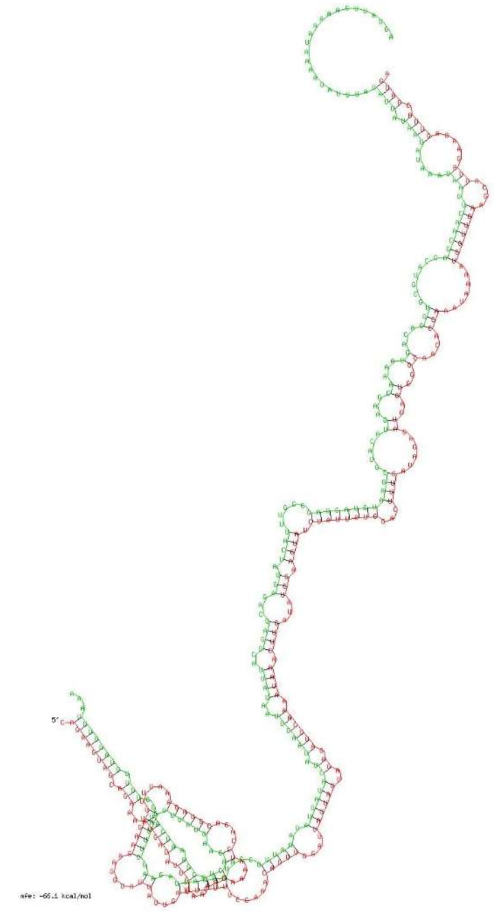


dltc:SprC

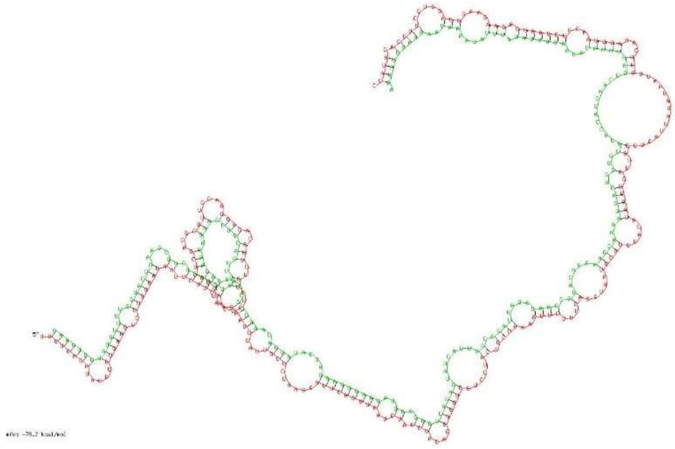


dps:SprC

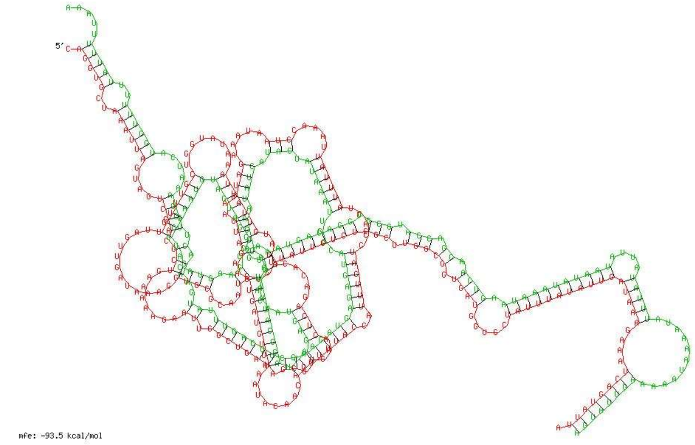


fruB:SprC

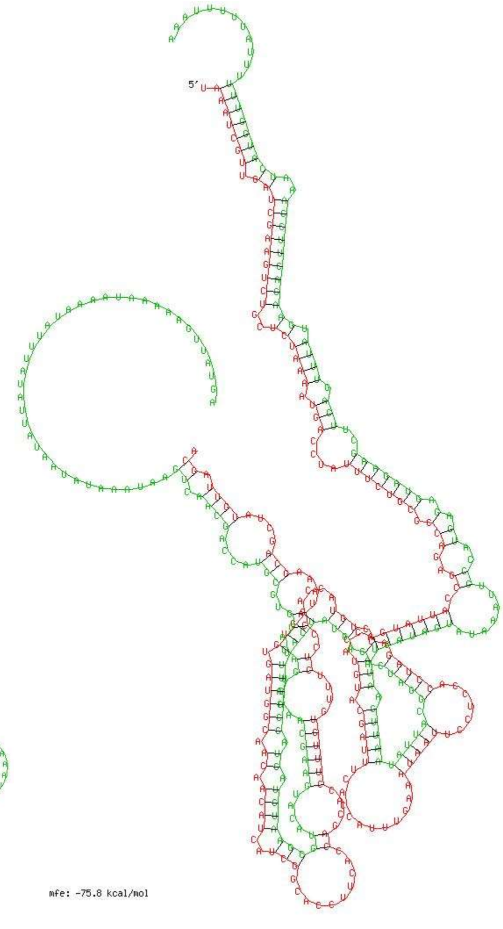


glcK:SprC

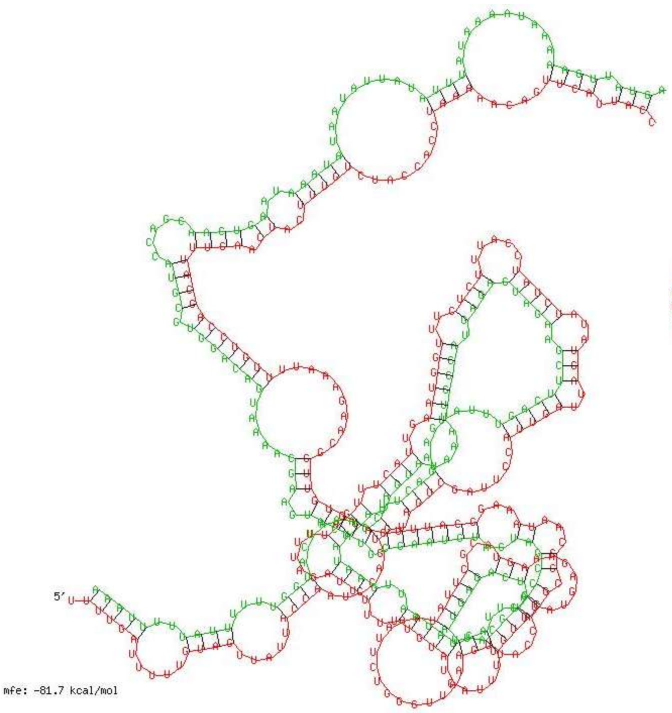


lukD:SprC

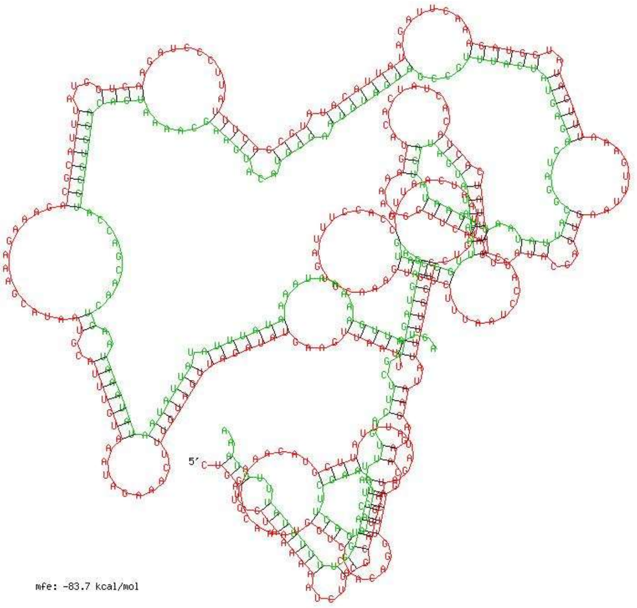


lukE:SprC

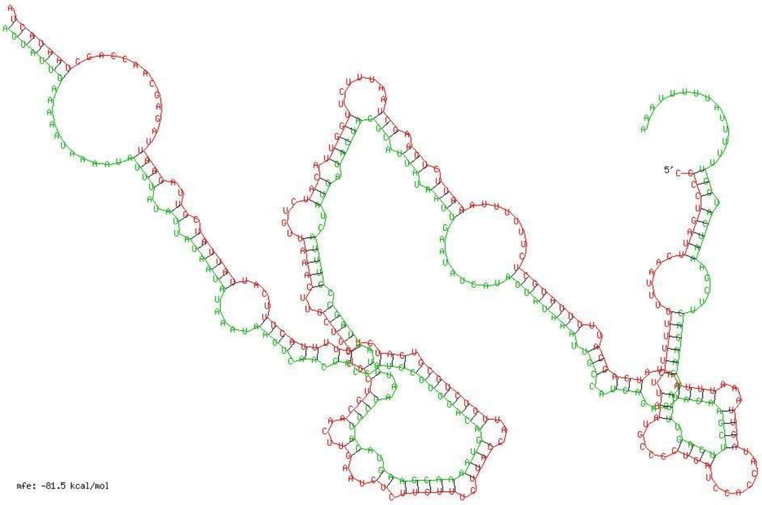


modA:SprC

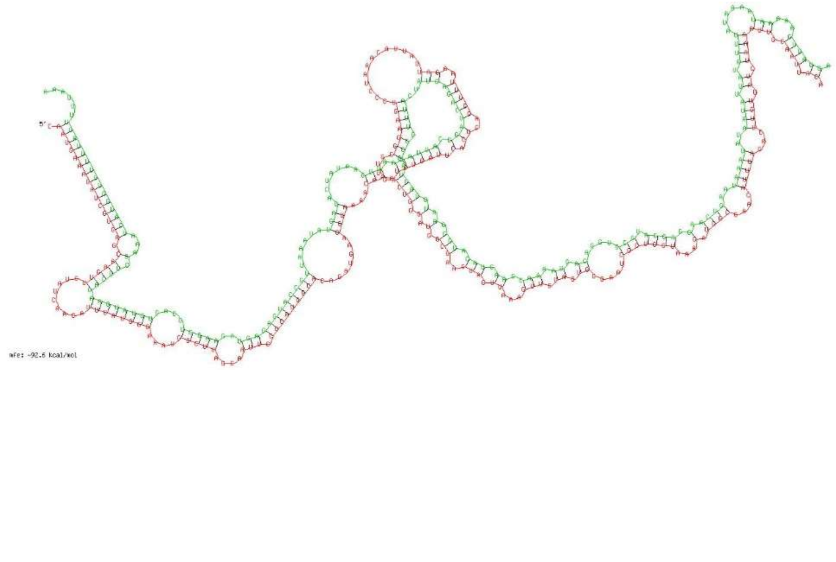


mtlA:SprC

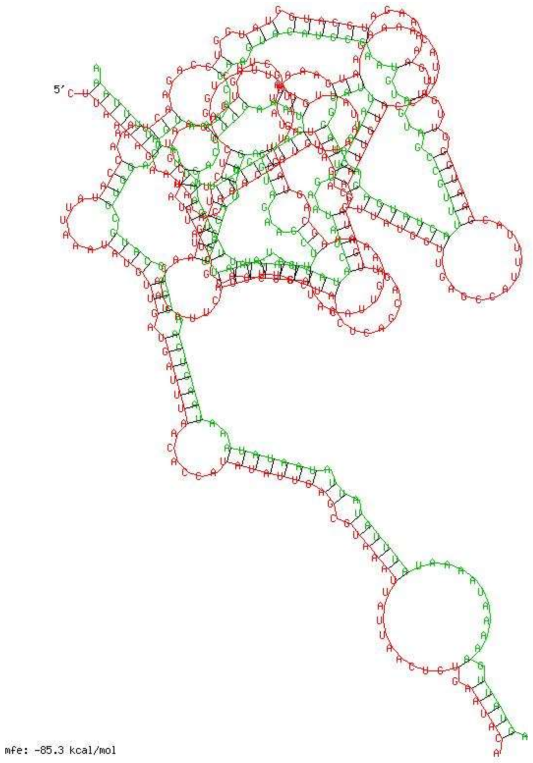


mtlD:SprC

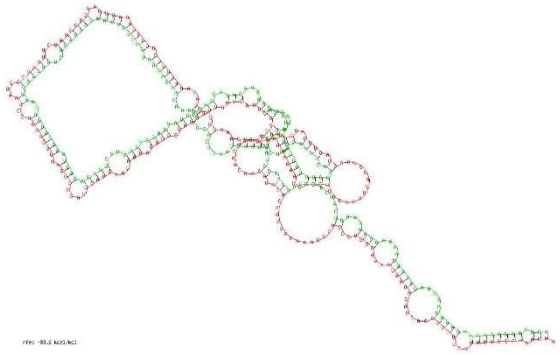

murG:SprC

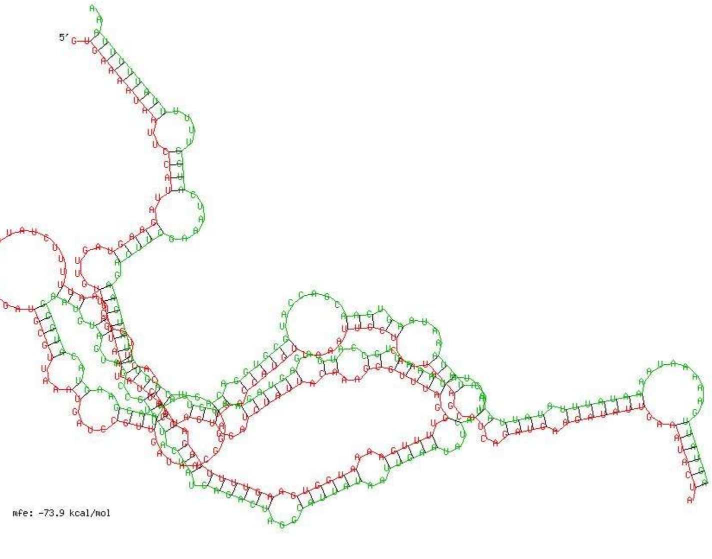


purC:SprC

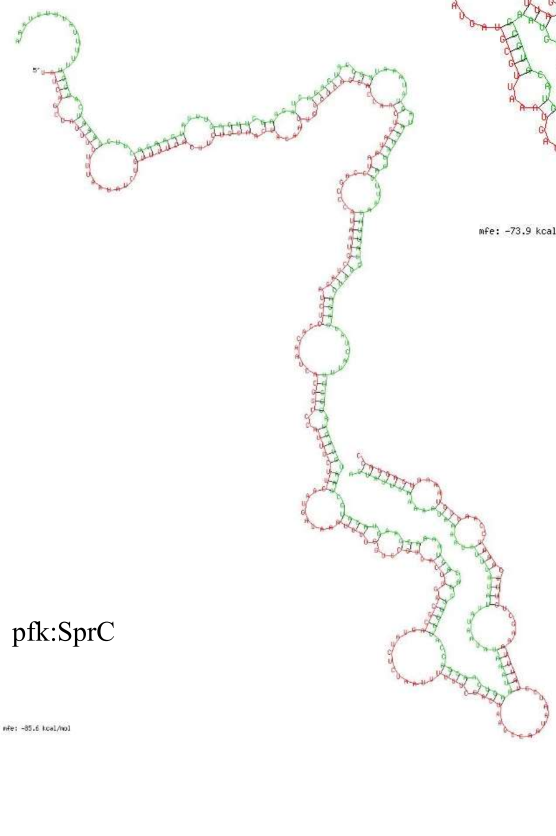

pfk:SprC

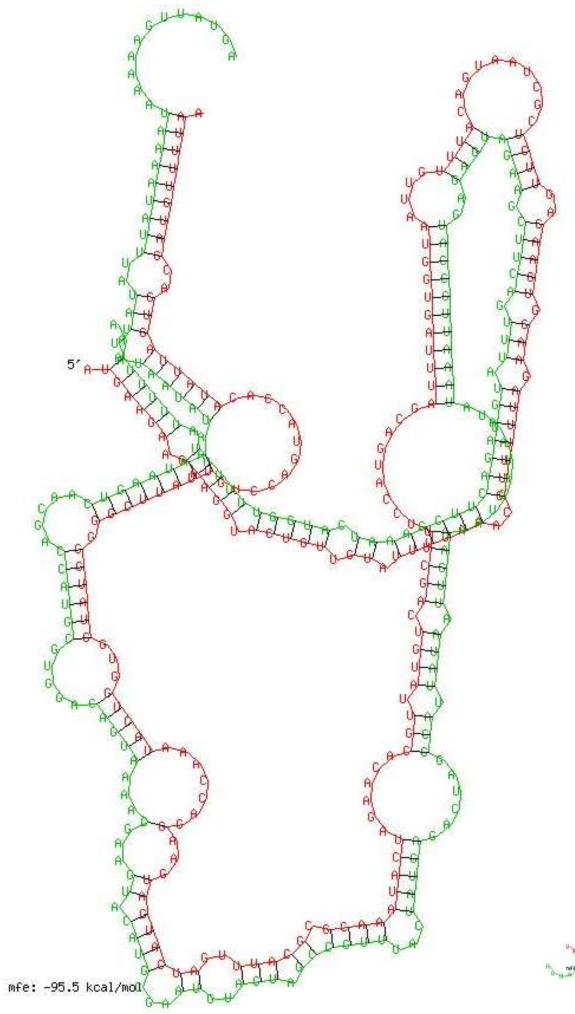


purD:SprC

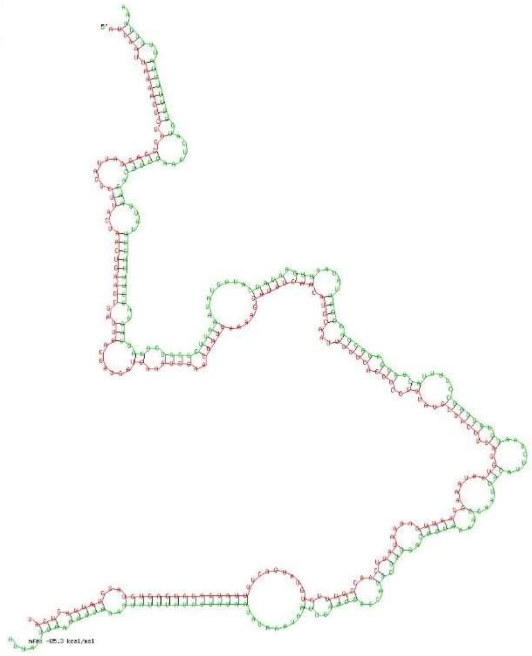

purF:SprC

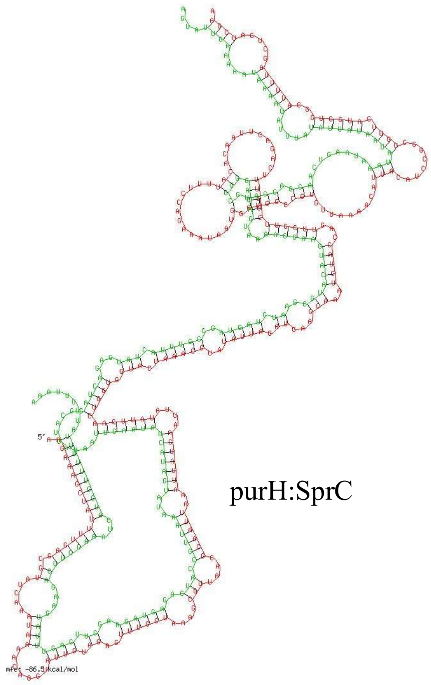

purH:SprC

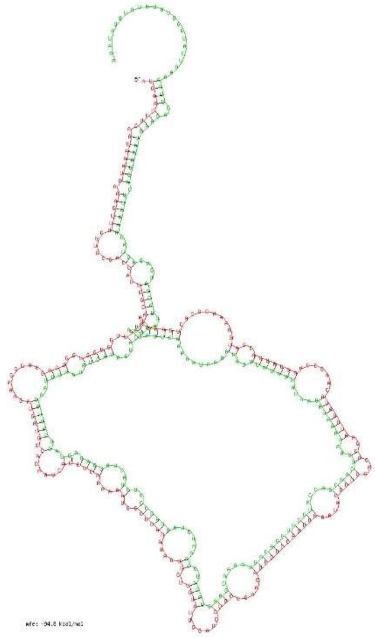

purK:SprC

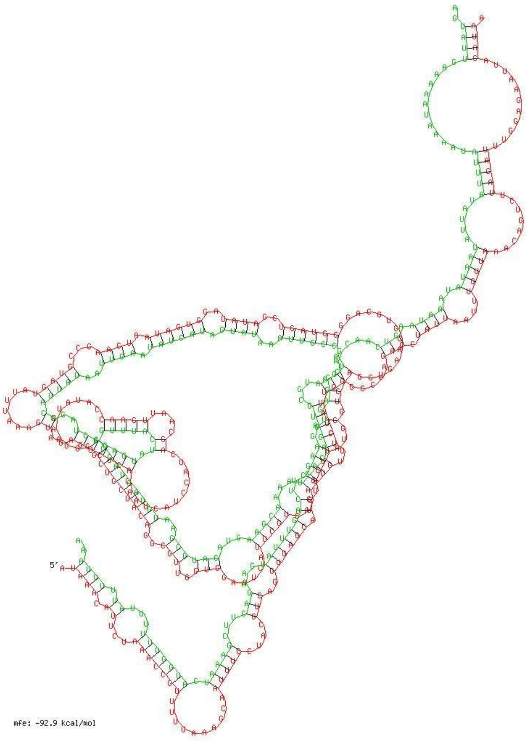


purL:SprC

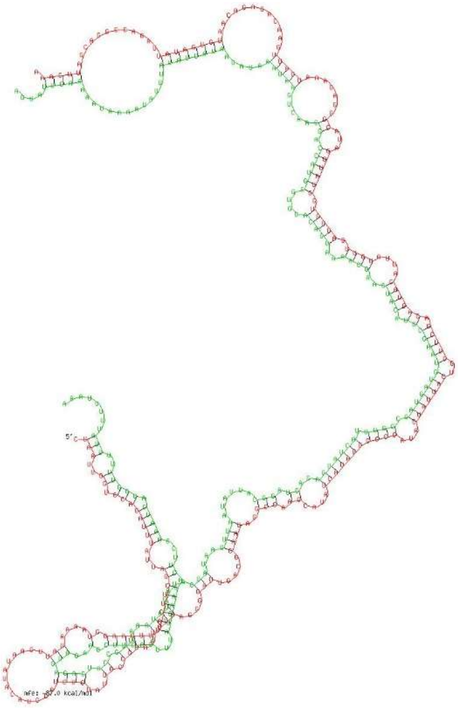


purN:SprC

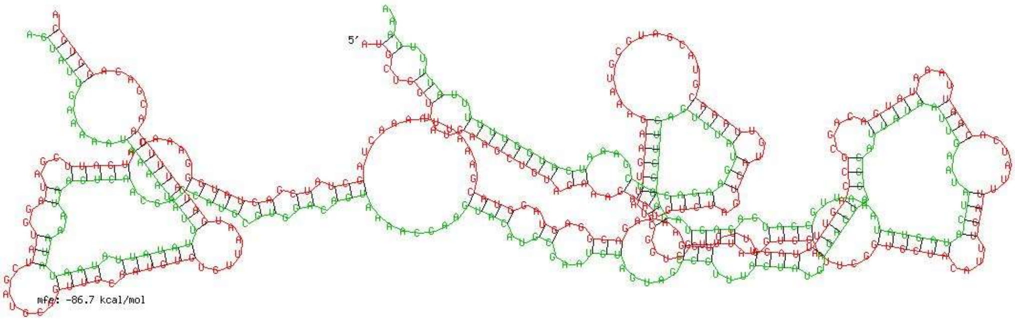


purM:SprC

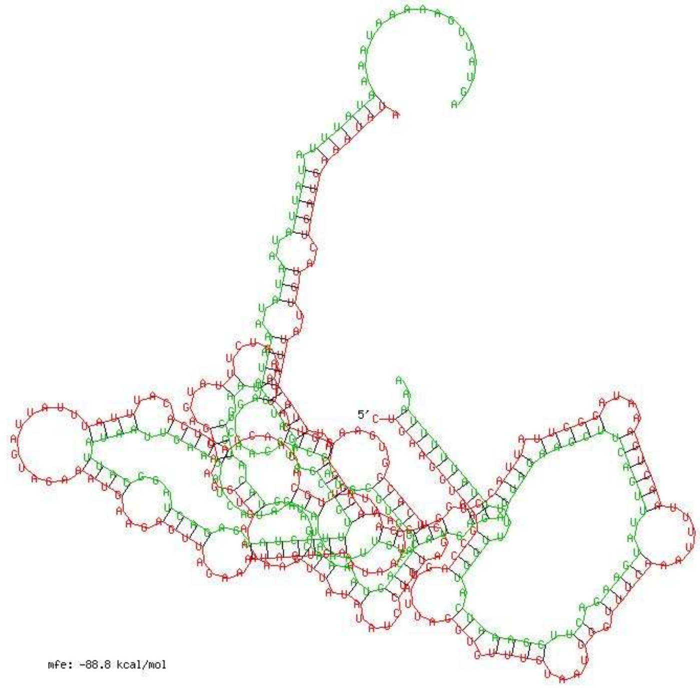


purQ:SprC

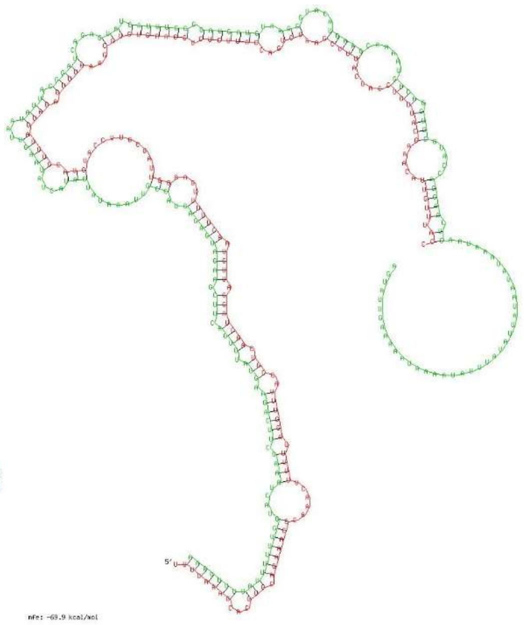

rpmB:SprC

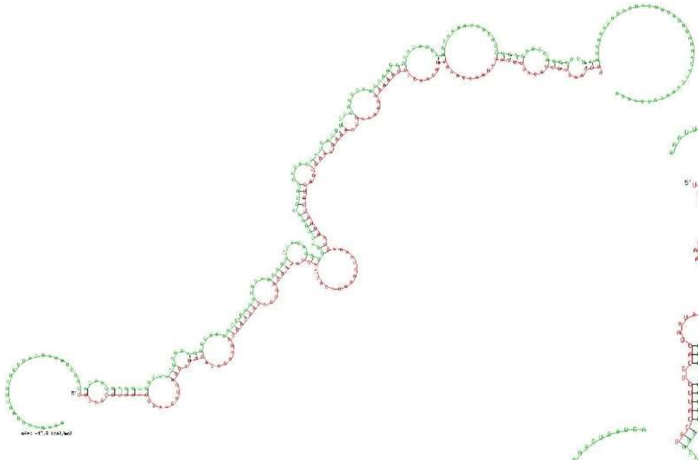

rpmG:SprC

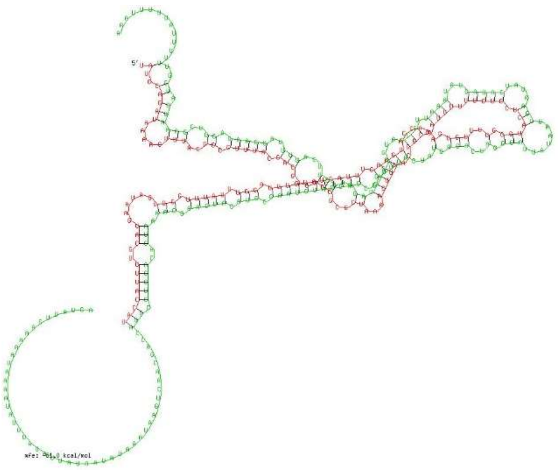

rpmH:SprC

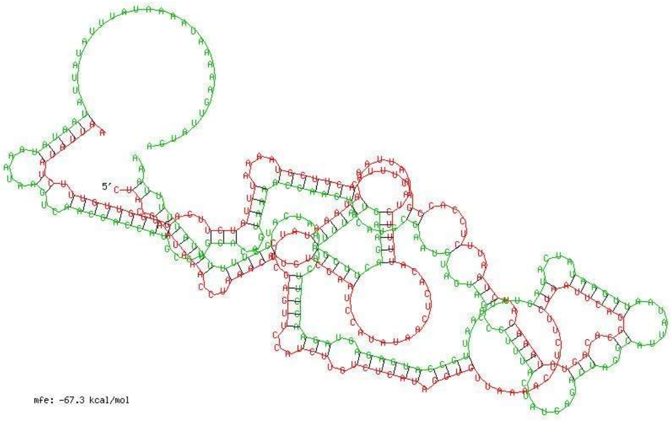


rsbV:SprC

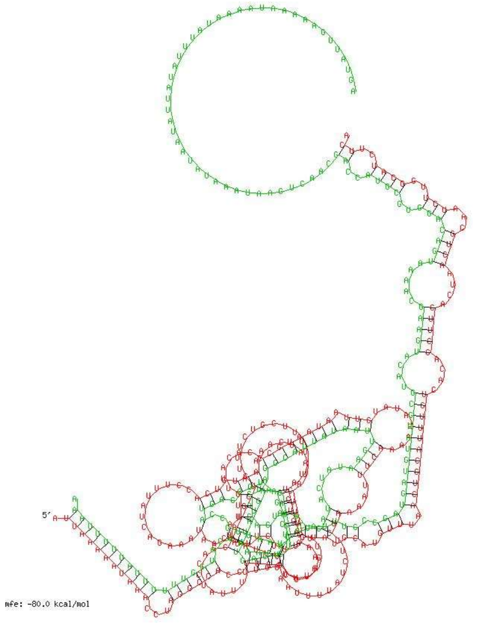


rsbW:SprC

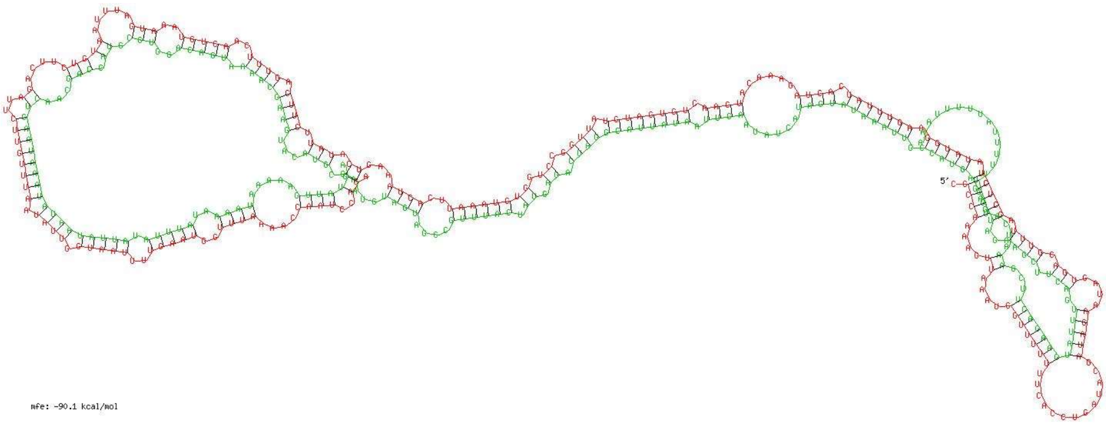


SA0231:SprC

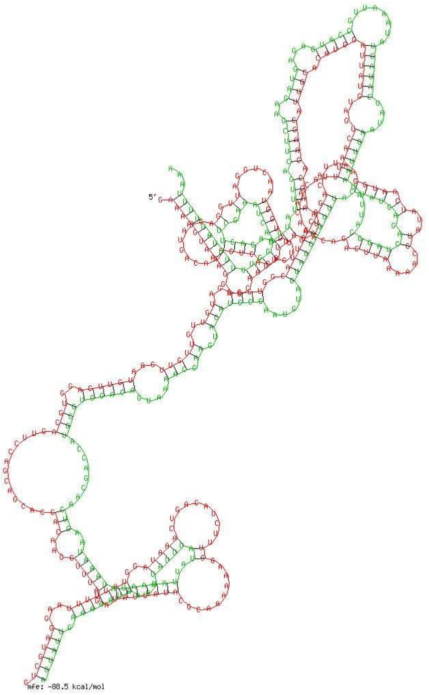

SA0239:SprC

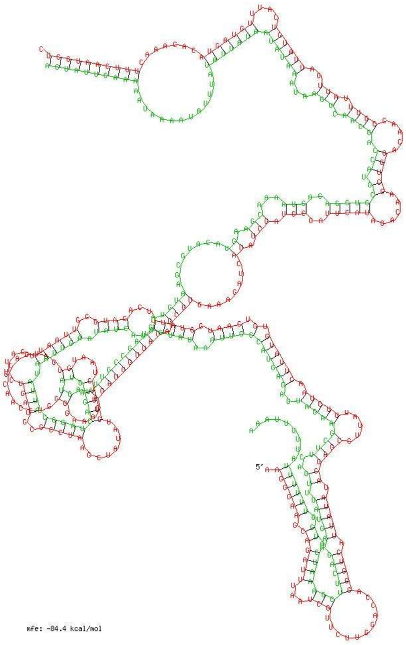

SA0721:SprC

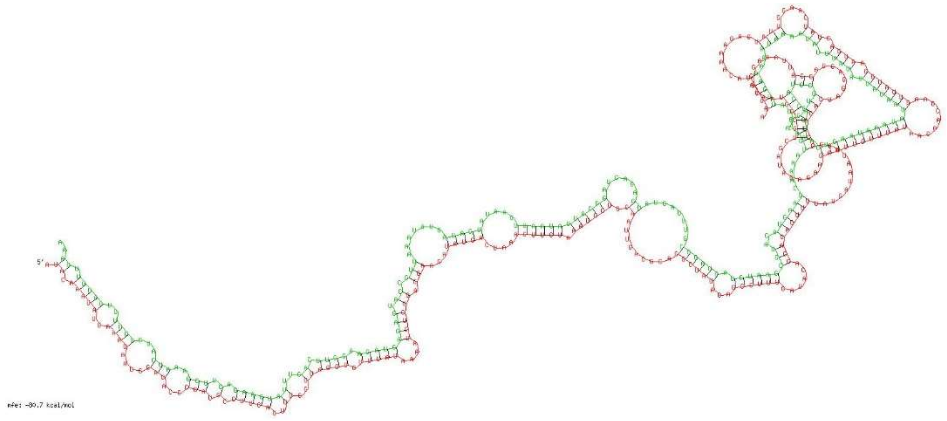

SA0658:SprC

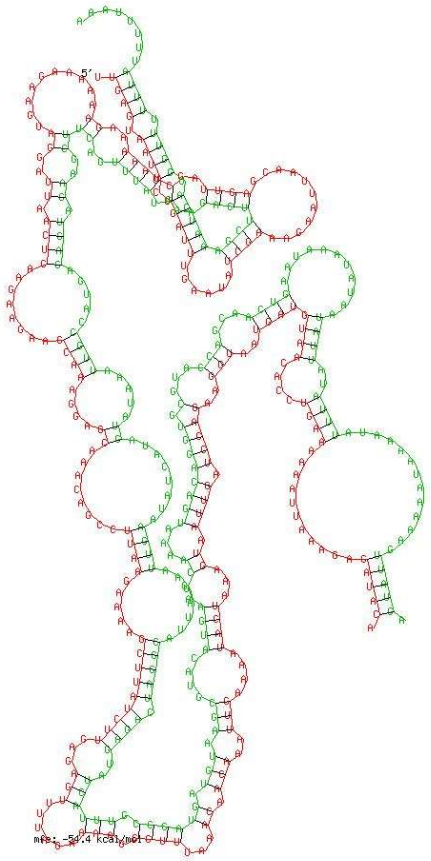

SA1176:SprC

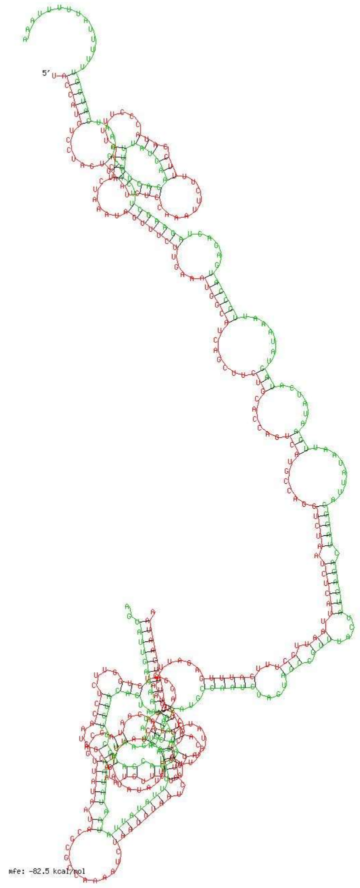


SA1360:SprC

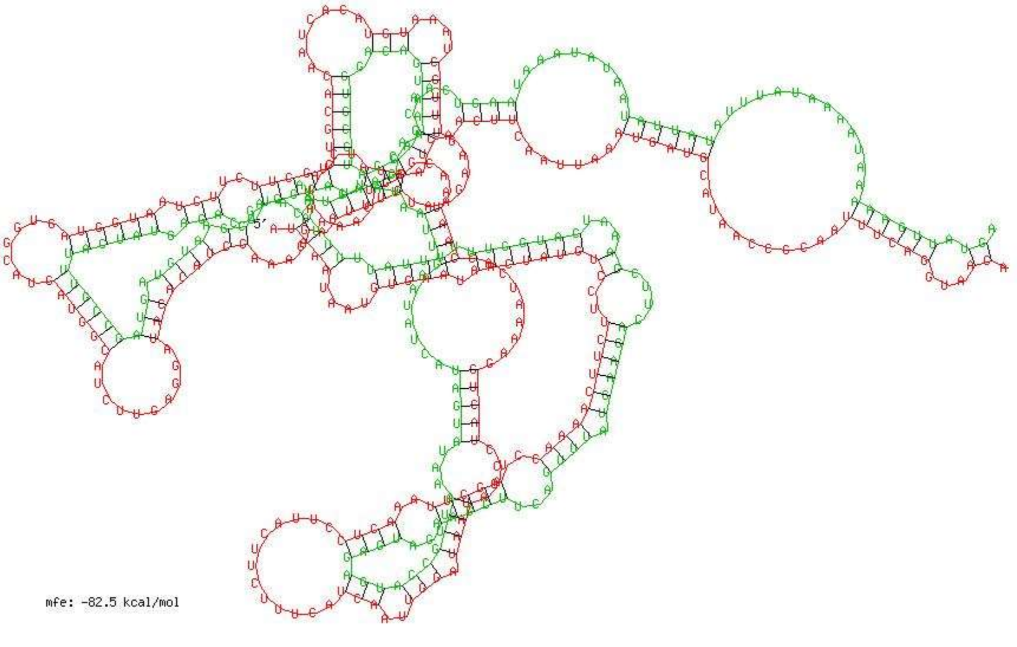


SA1534:SprC

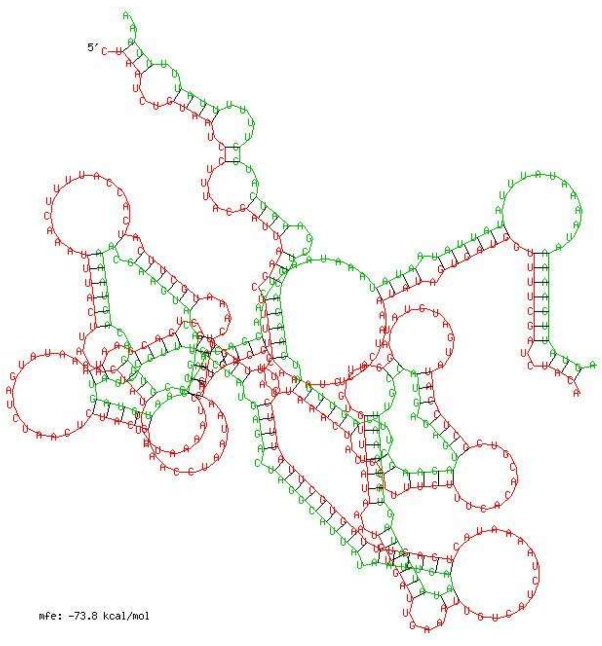


SA2297:SprC

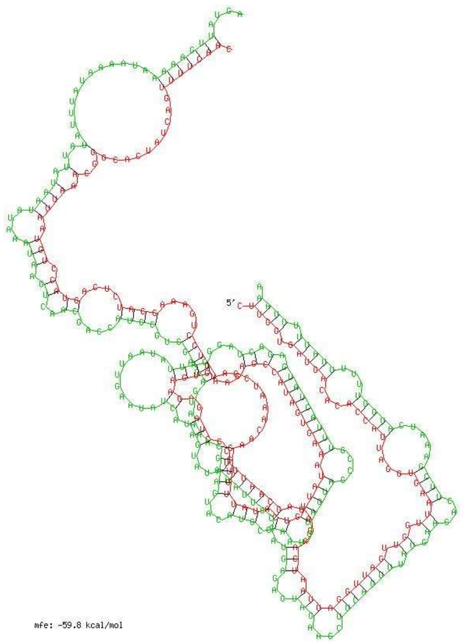


SA2331:SprC

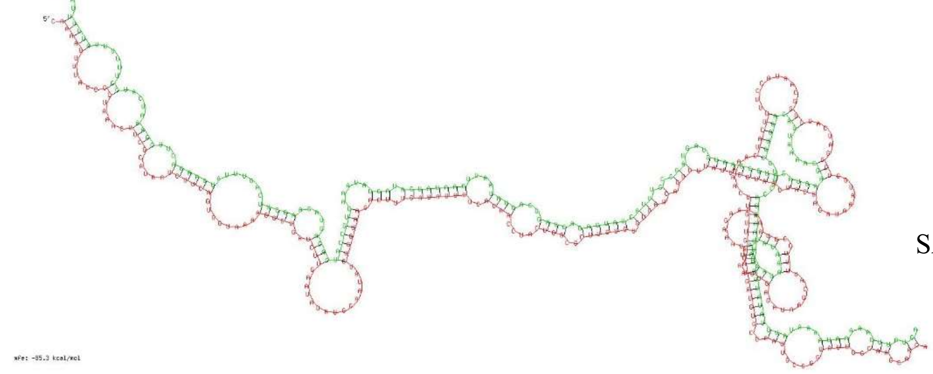

SA2351:SprC

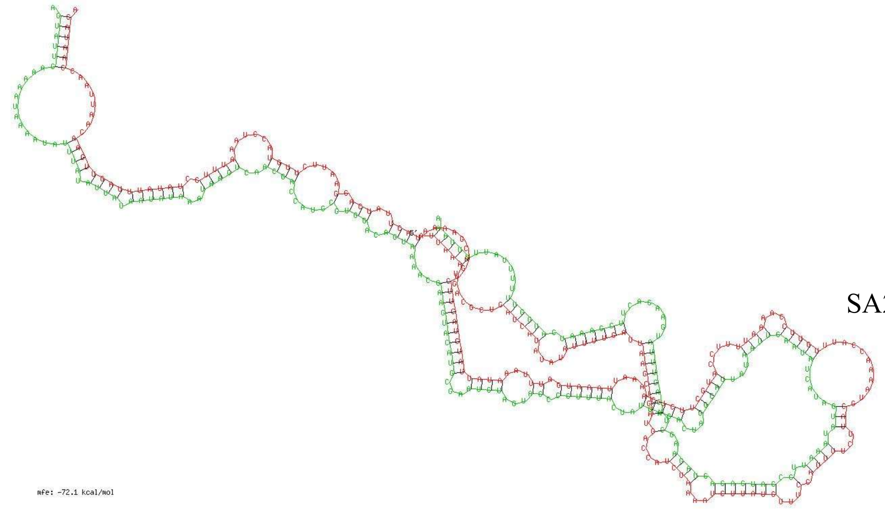


SA2352:SprC

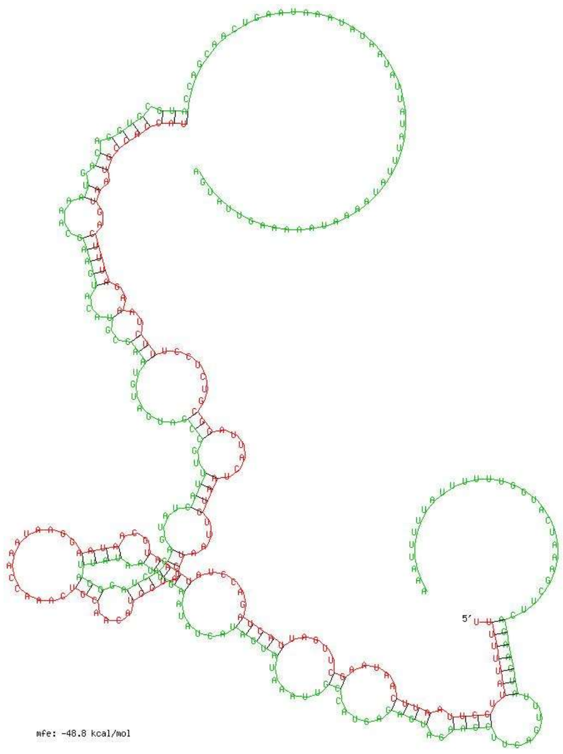


SAS059:SprC

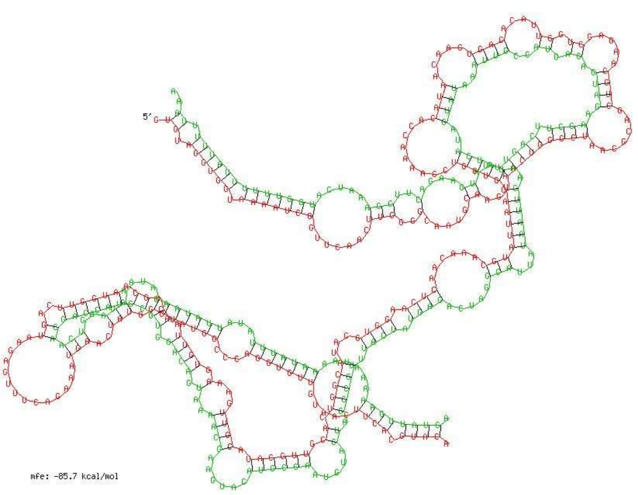


ssaA2:SprC

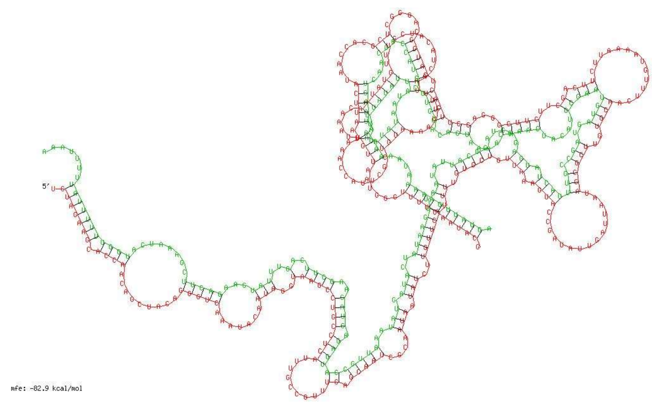


thiM:SprC

**Supplementary Figure 3.** Predicted secondary structure of mRNA of DEGs with defined function and SprC binding. The prediction was performed based on the bioinformatics tools on Bielefeld Bioinformatics Service (https://bibiserv.cebitec.uni-bielefeld.de/). We calculated the thermodynamic stability of SprC:mRNA duplex, with a standalone algorithm RNAhybrid. Molecular free energy (Mfe) of < 0 indicates that SprC and mRNA can bind spontaneously with

good affinity. Bases marked in red are SprC; green bases belong to DEGs.
