# Supplementary material for: Insights Into the Impact of Small RNA SprC on the Metabolism and Virulence of Staphylococcus aureus
Source: Front Cell Infect Microbiol. 2022 Feb 23;12:746746. doi: 10.3389/fcimb.2022.746746 (PMC8905650; doi:10.3389/fcimb.2022.746746)
Supplement: Supplementary Table 1 — The primer pairs used for the validation of DEGs expression. [file Table_1.docx]

**Supplementary Table 1. The primer pairs used for the validation of expression levels of the differentially expression genes (DEGs) from RNA-seq data (genes encoding hypothetical proteins excluded)**

| **Gene (ID)** | **Sequence of primer (5’ to 3’)** | **Size of production (bp)** | **Reference** |
| --- | --- | --- | --- |
| *lukE* (SAOUHSC_01955) | F: TCTCTTGCTGAACCTGTTGGACC | 197 | This study |
|  | R: AATGGCTCATTTAATTATTCTAA |  |  |
| *lukD* (SAOUHSC_01954) | F: AAAGGCATTTGATGTGTTGGCAA | 131 | This study |
|  | R: ATGAATAATGGTTGGGGACCATA |  |  |
| *rpmH* (SAOUHSC_03055) | F: TACGACGACGGCGCGCTAAAA | 75 | This study |
|  | R: ATAGTAAAGTTCATGGTTTCA |  |  |
| *ssaA2* (SAOUHSC_02571) | F: TGCAGGATTCGCTACAATCGC | 150 | This study |
|  | R: CCTTTCCATGTGTAATGGTAG |  |  |
| *purN* (SAOUHSC_01016) | F: GGTGGTTTAGGTGGATTCGGTGC | 142 | This study |
|  | R: CGATACCTATCGAATCATGTTTA |  |  |
| *purM* (SAOUHSC_01015) | F: GGTGGTTTAGGTGGATTCGGTGC | 366 | This study |
|  | R: AGCAAATCCAGCTACATCATATT |  |  |
| *mtlA* (SAOUHSC_02402) | F: CAGCCAAAATGAAGCAATTGA | 127 | This study |
|  | R: TAAGCCATTTCCCATAAATGT |  |  |
| *clpL* (SAOUHSC_02862) | F: CGCAAGAAGCACGTGACGGTTTA | 122 | This study |
|  | R: ACCAGCTTCTCCAACTAATATAG |  |  |
| *SA0239* (SAOUHSC_00217) | F: GTACATTATGCAGGAATTTGTGG | 219 | This study |
|  | R: GCATAAATTATAGTCTTTTGATT |  |  |
| *purF* (SAOUHSC_01014) | F: ATCCTGAAGCAGCGCAACTAACA | 103 | This study |
|  | R: CCTTTTAATTCATTTTGATCAGA |  |  |
| *ald* (SAOUHSC_01452) | F: GTCTTGGCGCTTTAGCACCTGGA  R: CAGATGTTACGATTTTAGATGTT | 171 | This study |
| *bex* (SAOUHSC_01668) | F: CGCATGAGGGATTTCTTCACTTG  R: ACCTAAATATTATCCAGATGATC | 112 | This study |
| *cspB* (SAOUHSC_03045) | F: TGGTTTAACGCAGAAAAAGGTTT  R: TGACCTTCAGTGATTTCGAAAGT | 143 | This study |
| *dltc* (SAOUHSC_00871) | F: TTAGCAGAAGTAGCAGAAAATGA  R: GAATCTCTAATAATAATCCAACT | 109 | This study |
| *dps* (SAOUHSC_02381) | F: GATTGCGTTTTCTAATTGTTTTG  R: AACCCTGTAGGTACATTAACTGA | 138 | This study |
| *fruB* (SAOUHSC_00707) | F: CTTGGGATTTGCAGGTGGATTTC  R: TTGATTTCTGTTTCTTGTCCTGT | 141 | This study |
| *glcK* (SAOUHSC_01646) | F: CGCCTGTCGCTGAAGCAACTGTT  R: CATCACACTTGGTACAGGTCTAG | 172 | This study |
| *modA* (SAOUHSC_02549) | F: TCTGATGTAGCACCAGCTTCGTA  R: CTGACTTATATAAACAAAACAAA | 100 | This study |
| *mtlD* (SAOUHSC_02403) | F: CCTTCTGAAGCGTTGAAACAAGC  R: CTCACAAGCAACAATATTAACAT | 144 | This study |
| *murG* (SAOUHSC_01424) | F: AAATCTTCTCGAATTGTTGCTCC  R: CAGCCAAATCATTAAATATACCA | 175 | This study |
| *pfk* (SAOUHSC_01807) | F: GTGCACCGCGATAACTACCGTCA  R: AAGAAGTACGTAAAGTTGCAATC | 93 | This study |
| *purC* (SAOUHSC_01010) | F: AGGAAAAGCGAAGCGCATTTTCT  R: CGTTTCCAGCAGTAACTTCATCT | 83 | This study |
| *purD* (SAOUHSC_01018) | F: CTGGTGGACGAGAACATGCACT  R: AATCTAGTATCGCTTGATGATC | 150 | This study |
| *purH* (SAOUHSC_01017) | F: ATTGTAGAGTTTGCTAAAGCGTT  R: CTGGAAAATGTGTTAAGTCTGAA | 127 | This study |
| *purK* (SAOUHSC_01009) | F: CAATCAGCTCAAAAAATGGGTTA  R: CGGAATATTGTACTTTTCACATA | 213 | This study |
| *purL* (SAOUHSC_01013) | F: GCCTGGTGAAGGTGCAGGGGTAG  R: TGGTTCAATTGCTGATGGATGAT | 94 | This study |
| *purQ* (SAOUHSC_01012) | F: TGCGGTTCTTGTTTTTCCAGGTT  R: ATAAGTACGCCATCAAATCCACT | 129 | This study |
| *rpmB* (SAOUHSC_01191) | F: GCACGTGCAGAAACCCAAACTT  R: TCACACGCTTTAAACTCTACTA | 104 | This study |
| *rpmG* (SAOUHSC_01328) | F: TGCGCGTAAACGTAACATT  R: CAATATTTTTTCATTTCAA | 106 | This study |
| *rsbV* (SAOUHSC_02300) | F: AACCTAAACCTGTCGAATCCAT  R: GGTGGAGAATTAGATGTTTATA | 130 | This study |
| *rsbW* (SAOUHSC_02299) | F: CACCTTCGCGTAAAAAGTCTAT  R: CAGTTAAACATGCATACAAAGA | 183 | This study |
| *SA0231* (SAOUHSC_00204) | F: AATGGCTTGCGATACTGTTGAA  R: CACACCTTTAGTAGCAATGTAT | 125 | This study |
| *SA0658* (SAOUHSC_00712) | F: CTTCAGTTGGGTTAGGTGTTTA  R: GAAGCCTCATTATCATAAAAGT | 121 | This study |
| *SA0721* (SAOUHSC_00788) | F: ACCAGCACCAGGAGACATCAGA  R: GAAATCATTCGTAATATTAGTC | 160 | This study |
| *SA1176* (SAOUHSC_01336) | F: TCAAGAAGAAGCAAAGGAGCAA  R: ATTACCTTCTGGATCAATTACT | 112 | This study |
| *SA1360* (SAOUHSC_01626) | F: ATTCTTATACCGCCCAAAC  R: AATGCTGGCTCGTACGATA | 96 | This study |
| *SA1534* (SAOUHSC_01821) | F: TTTGTTTCACCCGATTTTT  R: TTCTAGTGGTACCAAGTAA | 175 | This study |
| *SA2297* (SAOUHSC_02811) | F: ACTTTTCCGTCTCTGCATT  R: GTTGAGGTATTGCCTGTTG | 106 | This study |
| *SA2331* (SAOUHSC_02853) | F: TGCAAAGTTTTCTTGGTGA  R: TGTTCGAAAATATGACAAT | 119 | This study |
| *SA2351* (SAOUHSC_02881) | F: TTTCAATATCCTCCGGTGT  R: ATCCGACCATTTATCTAGT | 234 | This study |
| *SA2352* (SAOUHSC_02882) | F: AGCGTTGCACAATAATGAT  R: AGAGTTGATTCATTGGATA | 125 | This study |
| *SAS059* (SAOUHSC_02176) | F: TTTTTTATTGCTTAATTCA  R: AAATCTTTAGAAAGGAGAC | 117 | This study |
| *thiM* (SAOUHSC_02329) | F: TCATTTTCAGCAGCTACCTCAG  R: TGTTTATTAGGAGGCGTTATTG | 119 | This study |
| *16S RNA* | F: GCTGCCCTTTGTATTGTC | 179 | [Yang et al., 2020] |
|  | R: AGATGTTGGGTTAAGTCCC |  |  |
