# Supplementary material for: Insights Into the Impact of Small RNA SprC on the Metabolism and Virulence of Staphylococcus aureus
Source: Front Cell Infect Microbiol. 2022 Feb 23;12:746746. doi: 10.3389/fcimb.2022.746746 (PMC8905650; doi:10.3389/fcimb.2022.746746)
Supplement: Supplementary Table 2 — DEGs in N315ΔsprC and N315 strains. [file Table_2.docx]

**Supplementary Table 2. The differential expressed genes (DEGs) between N315 and N315Δ*sprC* mutant strains**

| **Gene ID** | **p-value** | **Log_2_FC** | **KEGG Pathway** | **Definition** | **Protein names** | **Molecular function** | **Biological process** | **Localization** |
| --- | --- | --- | --- | --- | --- | --- | --- | --- |
| SAOUHSC_00202 | 5.62E-08 | 1.03 |  | Hypothetical protein | Uncharacterized protein |  |  | Unknown |
| SAOUHSC_00204 | 0 | 1.20 |  | Globin domain-containing protein; K05916 nitric oxide dioxygenase | Nitric oxide dioxygenase | Oxidoreductase activity, heme binding, oxidoreductase activity | Oxidetion-reduction process | Unknown |
| SAOUHSC_00707 | 4.20E-82 | 1.14 | Fructose and mannose metabolism | Fructose 1-phosphate kinase; K00882 1-phosphofructokinase | Tagatose-6-phosphate kinase | Phosphotransferase activity, alcohol group as acceptor | Carbohydrate metabolic process | Unknown |
| SAOUHSC_00712 | 2.31E-29 | 1.15 |  | Hypothetical protein | Aldo_ket_red domain-containing protein | Oxidoreductase activity | Oxidation-reduction process | Unknown |
| SAOUHSC_00788 | 5.26E-45 | 1.10 |  | Hypothetical protein | Gluconeogenesis factor |  |  | Unknown |
| SAOUHSC_00871 | 2.71E-90 | 1.31 | D-Alanine metabolism, two-component system, cationic antimicrobial peptide (CAMP) resistance, Staphylococcus aureus infection | D-alanine—poly (phosphoribitol) ligase subunit 2; K14188 D-alanine—poly (phosphoribitol) ligase subunit 2 | D-alanyl carrier protein (DCP) (D-alanine—poly (phosphoribitol) ligase subunit 2) |  |  | Unknown |
| SAOUHSC_01009 | 1.19E-25 | 1.26 | Metabolic pathways, biosynthesis of secondary metabolites, biosynthesis of antibiotics, purine metabolism | Phosphoribosylaminoimidazole carboxylase ATPase subunit; K01589 5-(carboxyamino) imidazole ribonucleotide synthase | N5-carboxyaminoimidazole ribonucleotide synthase (N5-CAIR synthase) (5-(carboxyamino)imidazole ribonucleotide synthetase) | NAD binding, phosphoribosylaminoimidazole carboxylase | Oxidation-reduction process | Unknown |
| SAOUHSC_01010 | 1.09E-17 | 1.32 | Metabolic pathways, biosynthesis of secondary metabolites, biosynthesis of antibiotics, purine metabolism | Phosphoribosylaminoimidazole-succinocarboxamide synthase; K01923 phosphoribosylaminoimidazole-succinocarboxamide synthase | Phosphoribosylaminoimidazole-succinocarboxamide synthase (SAICAR synthetase) |  |  | Unknown |
| SAOUHSC_01012 | 2.99E-27 | 1.27 | Metabolic pathways, biosynthesis of secondary metabolites, biosynthesis of antibiotics, purine metabolism | Phosphoribosylformylglycinamidine synthase I; K01952 phosphoribosylformylglycinamidine synthase | Phosphoribosylformylglycinamidine synthase subunit PurQ (FGAM synthase) (Formylglycinamide ribonucleotide amidotransferase subunit I) (FGAR amidotransferase I) (FGAR-AT I) (Glutaminase PurQ) (Phosphoribosylformylglycinamidine synthase subunit I) |  |  | Unknown |
| SAOUHSC_01013 | 1.37E-141 | 1.55 | Metabolic pathways, biosynthesis of secondary metabolites, biosynthesis of antibiotics, purine metabolism | Phosphoribosylformylglycinamidine synthase II; K01952 phosphoribosylformylglycinamidine synthase | Phosphoribosylformylglycinamidine synthase subunit PurL (FGAM synthase) (Formylglycinamide ribonucleotide amidotransferase subunit II) (FGAR amidotransferase II) (FGAR-AT II) (Glutamine amidotransferase PurL) (Phosphoribosylformylglycinamidine synthase subunit II) |  |  | Unknown |
| SAOUHSC_01014 | 8.01E-126 | 1.25 | Metabolic pathways, biosynthesis of secondary metabolites, biosynthesis of antibiotics, purine metabolism, alanine, aspartate and glutamate metabolism | Amidophosphoribosyltransferase; K00764 amidophosphoribosyltransferase | Amidophosphoribosyltransferase (ATase) (Glutamine phosphoribosylpyrophosphate amidotransferase) (GPATase) |  | Nucleoside metabolic process | Unknown |
| SAOUHSC_01015 | 3.61E-58 | 1.49 | Metabolic pathways, biosynthesis of secondary metabolites, biosynthesis of antibiotics, purine metabolism | Phosphoribosylaminoimidazole synthetase; K01933 phosphoribosylformylglycinamidine cyclo-ligase | Phosphoribosylformylglycinamidine cyclo-ligase (AIR synthase) (AIRS) (Phosphoribosyl-aminoimidazole synthetase) |  |  | Unknown |
| SAOUHSC_01016 | 2.98E-47 | 1.56 | Metabolic pathways, biosynthesis of secondary metabolites, biosynthesis of antibiotics, purine metabolism, one carbon pool by folate | Phosphoribosylglycinamide formyltransferase; K11175 phosphoribosylglycinamide formyltransferase 1 | Phosphoribosylglycinamide formyltransferase (5'-phosphoribosylglycinamide transformylase) (GAR transformylase) (GART) |  |  | Unknown |
| SAOUHSC_01017 | 1.64E-257 | 1.75 | Metabolic pathways, biosynthesis of secondary metabolites, biosynthesis of antibiotics, purine metabolism, one carbon pool by folate | PurH; bifunctional phosphoribosylaminoimidazolecarboxamide formyltransferase/IMP cyclohydrolase; K00602 phosphoribosylaminoimidazolecarboxamide formyltransferase / IMP cyclohydrolase | Bifunctional purine biosynthesis protein PurH [Includes: Phosphoribosylaminoimidazolecarboxamide formyltransferase (AICAR transformylase); IMP cyclohydrolase (ATIC) (IMP synthase) (Inosinicase)] | Catalytic activity |  | Unknown |
| SAOUHSC_01018 | 0 | 1.14 | Metabolic pathways, biosynthesis of secondary metabolites, biosynthesis of antibiotics, purine metabolism | Phosphoribosylamine--glycine ligase; K01945 phosphoribosylamine--glycine ligase | Phosphoribosylamine--glycine ligase (GARS) (Glycinamide ribonucleotide synthetase) (Phosphoribosylglycinamide synthetase) |  |  | Unknown |
| SAOUHSC_01336 | 9.32E-11 | 1.13 |  | Hypothetical protein | UPF0291 protein |  |  | Unknown |
| SAOUHSC_01424 | 5.40E-18 | 1.01 | Peptidoglycan biosynthesis, vancomycin resistance, metabolic pathways | MurG; undecaprenyldiphospho-muramoylpentapeptide beta-N-acetylglucosaminyltransferase; K02563 UDP-N-acetylglucosamine--N-acetylmuramyl-(pentapeptide) pyrophosphoryl-undecaprenol N-acetylglucosamine transferase | UDP-N-acetylglucosamine--N-acetylmuramyl-(pentapeptide) pyrophosphoryl-undecaprenol N-acetylglucosamine transferase (Undecaprenyl-PP-MurNAc-pentapeptide-UDPGlcNAc GlcNAc transferase) |  |  | Unknown |
| SAOUHSC_01452 | 2.48E-17 | 1.15 | Taurine and hypotaurine metabolism, metabolic pathways, alanine, aspartate and glutamate metabolism | Alanine dehydrogenase; K00259 alanine dehydrogenase | Alanine dehydrogenase 1 | Oxidoreductase activity | Oxidation-reduction process | Unknown |
| SAOUHSC_01626 | 9.13E-48 | 1.03 |  | proline dipeptidase; K01262 Xaa-Pro aminopeptidase | Proline dipeptidase, putative | Hydrolase activity |  | Unknown |
| SAOUHSC_01646 | 8.45E-44 | 1.01 | Glycolysis / gluconeogenesis, starch and sucrose metabolism, streptomycin biosynthesis, amino sugar and nucleotide sugar metabolism, metabolic pathways, biosynthesis of secondary metabolites, biosynthesis of antibiotics, microbial metabolism in diverse environments, carbon metabolism, galactose metabolism | Glucokinase; K00845 glucokinase | Glucokinase (Glucose kinase) |  |  | Unknown |
| SAOUHSC_01668 | 1.31E-37 | 1.06 |  | Era; GTP-binding protein Era; K03595 GTPase | GTPase Era | RNA binding, GTP binding |  | Intracellular |
| SAOUHSC_01807 | 2.12E-205 | 1.14 | Glycolysis / gluconeogenesis, methane metabolism, Pentose phosphate pathway, biosynthesis of amino acids, RNA degradation, metabolic pathways, biosynthesis of secondary metabolites, biosynthesis of antibiotics, fructose and mannose metabolism, microbial metabolism in diverse environments, carbon metabolism, galactose metabolism | 6-phosphofructokinase; K00850 6-phosphofructokinase 1 | ATP-dependent 6-phosphofructokinase (ATP-PFK) (Phosphofructokinase) (Phosphohexokinase) |  |  | Unknown |
| SAOUHSC_01821 | 2.52E-37 | 1.09 |  | Hypothetical protein; K00571 site-specific DNA-methyltransferase (adenine-specific) | N6_Mtase domain-containing protein | DNA binding, N-methyltransferase activity | DNA methylation | Unknown |
| SAOUHSC_02299 | 1.21E-45 | 1.16 |  | Serine-protein kinase RsbW; K04757 serine/threonine-protein kinase RsbW | Serine-protein kinase RsbW (Anti-sigma-B factor) (Sigma-B negative effector RsbW) | Protein serine/threonine kinase activity, ATP binding, sigma factor antagonist activity | Protein phosphorylation, negative regulation of transcription, DNA-templated | Unknown |
| SAOUHSC_02300 | 3.01E-24 | 1.01 |  | STAS domain-containing protein; K04749 anti-sigma B factor antagonist | Anti-sigma-B factor antagonist (Anti-anti-sigma-B factor) | Antisigma factor binding | Regulation of transcription, DNA-templated | Unknown |
| SAOUHSC_02329 | 1.14E-09 | 1.02 | Metabolic pathways, thiamine metabolism | Hydroxyethylthiazole kinase; K00878 hydroxyethylthiazole kinase | Hydroxyethylthiazole kinase (4-methyl-5-beta-hydroxyethylthiazole kinase) (TH kinase) (Thz kinase) |  |  | Unknown |
| SAOUHSC_02402 | 1.30E-11 | 1.38 | Phosphotransferase system (PTS), fructose and mannose metabolism | PTS system mannitol-specific transporter subunit IIA; K02798 PTS system, mannitol-specific IIA component | EIIA (EIII) (Mannitol-specific phosphotransferase enzyme IIA component) (PTS system mannitol-specific EIIA component) |  |  | Unknown |
| SAOUHSC_02403 | 2.20E-21 | 1.05 | Fructose and mannose metabolism | Mannitol-1-phosphate 5-dehydrogenase; K00009 mannitol-1-phosphate 5-dehydrogenase | Mannitol-1-phosphate 5-dehydrogenase | Oxidoreductase activity, coenzyme binding | Oxidation-reduction process | Unknown |
| SAOUHSC_02442 | 1.39E-192 | 1.12 |  | Hypothetical protein | Uncharacterized protein |  |  | Unknown |
| SAOUHSC_02448 | 1.77E-18 | 1.11 |  | Hypothetical protein | Uncharacterized protein |  |  | Unknown |
| SAOUHSC_02549 | 2.57E-45 | 1.07 | ABC transporters, | Molybdenum ABC transporter substrate-binding protein; K02020 molybdate transport system substrate-binding protein | Molybdenum ABC transporter, periplasmic molybdate-binding protein | Molybdate transmembrane-transporting ATPase activity | Molybdate ion transport | Outer membrane-bounded periplasmic space |
| SAOUHSC_02811 | 4.86E-15 | 1.22 | Purine metabolism | Hypothetical protein; K07816 putative GTP pyrophosphokinase | RelA_SpoT domain-containing protein |  | Guanosine tetraphosphate metabolic process | Unknown |
| SAOUHSC_02862 | 0 | 1.38 |  | ATP-dependent Clp protease ATP-binding subunit ClpC; K04086 ATP-dependent Clp protease ATP-binding subunit ClpL | ATP-dependent Clp protease ATP-binding subunit ClpL | Protein binding, ATP binding |  | Unknown |
| SAOUHSC_02881 | 3.55E-18 | 1.02 | Carotenoid biosynthesis | Hypothetical protein; K10210 diapolycopene oxygenase | 4,4'-diaponeurosporene oxygenase (4,4'-diaponeurosporene oxidase) (Carotenoid oxidase) | Oxidoreductase activity | Oxidation-reduction process, carotenoid biosynthetic process | Unknown |
| SAOUHSC_02882 | 7.21E-08 | 1.12 | Carotenoid biosynthesis | Hypothetical protein; K10212 glycosyl-4,4'-diaponeurosporenoate acyltransferase | Glycosyl-4,4'-diaponeurosporenoate acyltransferase |  |  | Unknown |
| SAOUHSC_02896 | 0.000272 | 1.18 |  | hypothetical protein | Uncharacterized protein |  |  | Unknown |
| SAOUHSC_02906 | 0.001806 | 1.02 |  | hypothetical protein | Uncharacterized protein |  |  | Unknown |
| SAOUHSC_00217 | 4.12E-07 | -1.37 | Pentose and glucuronate interconversions, metabolic pathways, fructose and mannose metabolism | Sorbitol dehydrogenase; K00008 L-iditol 2-dehydrogenase | Alcohol dehydrogenase | Zinc ion binding, oxidoreductase activity | Oxidation-reduction process | Unknown |
| SAOUHSC_00473 | 3.65E-71 | -1.03 |  | Hypothetical protein | Uncharacterized protein |  |  | Unknown |
| SAOUHSC_00966 | 2.69E-84 | -1.01 |  | Hypothetical protein | Uncharacterized protein |  |  | Unknown |
| SAOUHSC_00971 | 2.69E-55 | -1.02 |  | Hypothetical protein | Uncharacterized protein |  |  | Unknown |
| SAOUHSC_00975 | 0 | -1.17 |  | Hypothetical protein; K15977 putative oxidoreductase | Uncharacterized protein |  |  | Unknown |
| SAOUHSC_01191 | 0 | -1.06 | Ribosome | RpmB; 50S ribosomal protein L28; K02902 large subunit ribosomal protein L28 | 50S ribosomal protein L28 | Structural constituent of ribosome | Translation | Intracellular, ribosome |
| SAOUHSC_01328 | 1.68E-169 | -1.15 | Ribosome | RpmG; 50S ribosomal protein L33; K02913 large subunit ribosomal protein L33 | 50S ribosomal protein L33 | Structural constituent of ribosome | Translation | Intracellular, ribosome |
| SAOUHSC_01954 | 3.68E-153 | -5.15 | *Staphylococcus aureus* infection | Leukotoxin LukD; K11038 leukocidin/hemolysin toxin family protein | Leucotoxin LukDv (variant of LukD) |  | Cytolysis in other organism, pathogenesis | Extracellular region |
| SAOUHSC_01955 | 4.51E-134 | -4.98 | *Staphylococcus aureus* infection | Leukotoxin LukE; K11038 leukocidin/hemolysin toxin family protein | Leucotoxin LukEv (variant of LukE) |  | Cytolysis in other organism, pathogenesis | Extracellular region |
| SAOUHSC_02175 | 4.35E-257 | -1.10 |  | Hypothetical protein | Hypothetical phage protein |  |  | Unknown |
| SAOUHSC_02176 | 0 | -1.07 |  | Hypothetical protein | Conserved hypothetical phage protein |  |  | Unknown |
| SAOUHSC_02294 | 1.67E-34 | -1.03 |  | Hypothetical protein | Uncharacterized protein |  |  | Unknown |
| SAOUHSC_02381 | 0 | -1.05 |  | Hypothetical protein; K04047 starvation-inducible DNA-binding protein | Ferritin domain-cotaining protein | Oxidoreductase activity, oxidizing metal ions, ferric iron binding | Oxidation-reduction process, cellular iron ion homeostasis | Unknown |
| SAOUHSC_02391 | 0 | -1.03 |  | Hypothetical protein | Uncharacterized protein |  |  | Unknown |
| SAOUHSC_02411 | 1.46E-18 | -2.03 |  | Hypothetical protein | Uncharacterized protein |  |  | Unknown |
| SAOUHSC_02412 | 0 | -1.87 |  | Hypothetical protein | Uncharacterized protein |  |  | Unknown |
| SAOUHSC_02521 | 7.59E-11 | -1.02 |  | Hypothetical protein | Uncharacterized protein |  |  | Unknown |
| SAOUHSC_02571 | 0 | -1.15 |  | Secretory antigen | Staphylococcal secretory antigen ssaA2 |  |  | Unknown |
| SAOUHSC_02702 | 9.40E-30 | -1.25 |  | Hypothetical protein | Uncharacterized protein |  |  | Unknown |
| SAOUHSC_02721 | 1.66E-67 | -1.56 |  | Hypothetical protein | Uncharacterized protein |  |  | Unknown |
| SAOUHSC_02853 | 2.32E-137 | -1.07 |  | Hypothetical protein | YozE_SAM_like domain-containing protein |  |  | Unknown |
| SAOUHSC_03045 | 0 | -1.01 |  | Cold shock protein; K03704 cold shock protein (beta-ribbon, CspA family) | Cold shock protein CspA | DNA binding, nucleic acid binding | Regulation of transcription, DNA-templated | Unknown |
| SAOUHSC_03055 | 0 | -1.17 | Ribosome | RpmH; 50S ribosomal protein L34; K02914 large subunit ribosomal protein L34 | 50S ribosomal protein L34 | Structural constituent of ribosome | translation | Intracellular, ribosome |
